# Transverse relaxation optimized spectroscopy of NH_2_ groups in glutamine and asparagine side chains of proteins

**DOI:** 10.1007/s10858-024-00445-8

**Published:** 2024-07-31

**Authors:** Vitali Tugarinov, Francesco Torricella, Jinfa Ying, G. Marius Clore

**Affiliations:** Laboratory of Chemical Physics, National Institute of Diabetes and Digestive and Kidney Diseases, National Institutes of Health, Bethesda, MD 20892-0520 USA

**Keywords:** Cross correlated relaxation in NH_2_ groups, Transverse relaxation optimized spectroscopy (TROSY), ^15^N CPMG relaxation dispersion, Chemical exchange

## Abstract

A transverse relaxation optimized spectroscopy (TROSY) approach is described for the optimal detection of NH_2_ groups in asparagine and glutamine side chains of proteins. Specifically, we have developed NMR experiments for isolating the slow-relaxing ^15^N and ^1^H components of NH_2_ multiplets. Although even modest sensitivity gains in 2D NH_2_-TROSY correlation maps compared to their decoupled NH_2_–HSQC counterparts can be achieved only occasionally, substantial improvements in resolution of the NMR spectra are demonstrated for asparagine and glutamine NH_2_ sites of a buried cavity mutant, L99A, of T4 lysozyme at 5 ºC. The NH_2_-TROSY approach is applied to CPMG relaxation dispersion measurements at the side chain NH_2_ positions of the L99A T4 lysozyme mutant — a model system for studies of the role of protein dynamics in ligand binding.

**Supplementary Information:**

The online version contains supplementary material available at 10.1007/s10858-024-00445-8.

## Introduction

Transverse relaxation optimized spectroscopy (TROSY) has had a transformative impact on solution NMR studies of high molecular weight biological macromolecules and biomolecular assemblies. Developed originally for ^15^N-^1^H spin-systems of backbone amides and tryptophan indole rings (Pervushin et al. 1997, 1998b, 2002; Salzmann et al. 1998; Riek et al. 2002; Yang and Kay 1999a, 1999b) and ^13^C–^1^H spin-systems in aromatic rings of proteins (Pervushin et al. 1998a), the principles of TROSY were later extended to ^13^C–^1^H_3_ (Tugarinov et al. 2003; Ollerenshaw et al. 2003) and ^13^C–^1^H_2_D (Tugarinov et al. 2005) methyl groups in protein side chains, as well as ^13^C–^1^H_2_ spin-systems in proteins and nucleic acids (Miclet et al. 2004). Relaxation optimized NMR experiments have been developed for lysine ^15^N–^1^H_3_ groups (Iwahara et al. 2007), and ^13^C–^19^F spin pairs in aromatic rings of proteins (Boeszoermenyi et al. 2019). While the optimization of transverse relaxation in TROSY experiments is generally achieved through isolation of the slow-relaxing components of spin-system multiplets, decreases in transverse relaxation rates result from destructive interference of random, fluctuating magnetic fields that lie at the source of decay of nuclear magnetization (Goldman 1984). Depending on the type of spin-system in question, such interference may occur between the magnetic fields experienced by different dipoles (dipole–dipole cross-correlated relaxation, DD) or between dipoles and the chemical shielding anisotropy (CSA) of a given nucleus (CSA/DD cross-correlated relaxation).

Here we apply the principles of TROSY to carboxamide NH_2_ groups in asparagine and glutamine side chains of proteins. We identify ^15^N and ^1^H slow-relaxing components of the NH_2_ multiplets and describe NMR experiments for their isolation. TROSY effects in carboxamide NH_2_ groups are compared with those in ^15^N–^1^H and ^13^C–^1^H_2_ spin-systems. Although NMR experiments for simultaneous detection of backbone ^15^N–^1^H, aromatic ^13^C–^1^H and side chain ^15^N–^1^H_2_ correlations in high molecular weight proteins were described earlier (Pervushin et al. 2000), these experiments were not optimized specifically for NH_2_ groups—particularly, the ^1^H transitions of ^15^N–^1^H_2_ spin-systems. Notwithstanding that sensitivity gains in 2D NH_2_-TROSY correlation maps compared to their (decoupled) heteronuclear single-quantum correlation (HSQC) (Bodenhausen and Ruben 1980) counterparts can be achieved only occasionally, substantial improvements in resolution of the NMR spectra are demonstrated for Asn and Gln NH_2_ groups in a buried cavity mutant, L99A, of T4 lysozyme (Eriksson et al. 1992b, 1992a) at 5 ºC. The developed NH_2_-TROSY approach is applied to Carr-Purcell-Meiboom-Gill (CPMG) (Carr and Purcell 1954; Meiboom and Gill 1958) relaxation dispersion measurements for the studies of chemical exchange on the μs-to-ms timescale at the side chain NH_2_ positions of the T4 lysozyme L99A mutant.

## Results and discussion

### Theoretical background

**Fig. 1:**
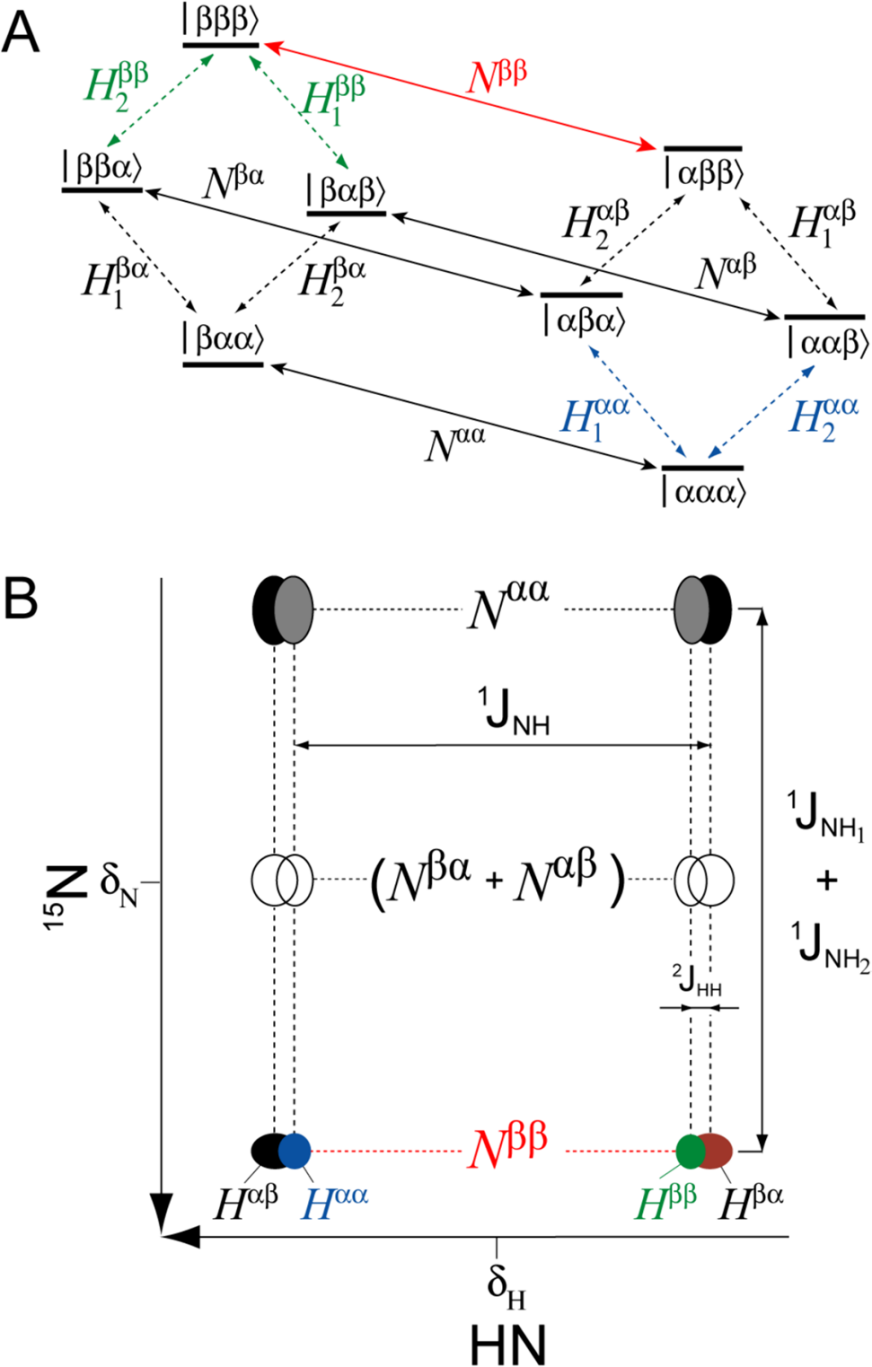
NMR transitions in an ^15^N–^1^H_2_ spin-system. **A** Energy level diagram of an isolated AMX (^15^N^1^H_1_^1^H_2_) spin system (NH_2_ group). Three symbols, ‘α’ or ‘β’, label each eigenstate, with the first symbol corresponding to the state of ^15^N, and the last two symbols, to the spin states of hydrogens H_1_ and H_2_. Diagonal solid arrows depict ^15^N transitions, each labeled with the single-transition operator defined in Eq. 1 and the Supplementary Information, SI, Eq. S1. Diagonal dashed arrows depict ^1^H transitions labeled with single-transition operators defined in Eq. 4 and SI, Eq. S4. The ^15^N transition associated with the slowest transverse relaxation rate and selected for in NH_2_-TROSY experiments is colored in red. The ^1^H transitions usually associated with the slowest transverse relaxation rates are colored in green, while those that can occasionally be associated with the slowest rates, are shown in blue. **B** Schematic representation of the 2D multiplet pattern observed for an individual ^1^H spin of an ^15^N–^1^H_2_ spin-system in the HSQC experiment performed without de-coupling in either dimension. The pattern is drawn for ^1^*J*_NH_ < 0, ^2^*J*_HH_ > 0, and ^1^*J*_NH1_ = ^1^*J*_NH2_. For ^1^*J*_NH1_ = ^1^*J*_NH2_, the intensities of ^15^N transitions *N*^αβ^ and *N*^βα^ vanish (shown with open circles). The frequencies of ^15^N and ^1^H nuclei are labeled with δ_N_ and δ_H_, respectively. Horizontal, short-dashed and vertical, long-dashed lines depict ^15^N and ^1^H transitions, respectively. The transverse relaxation rate of each ^15^N transition is defined in Eqs. 2, 3 and SI, Eqs. S2–S3, while that of each ^1^H transition, in Eqs. 5, 6 and SI, Eqs. S5–S6. The ^15^N transition associated with the slowest transverse relaxation rate is colored in red, while the ^1^H peaks usually (*H*^ββ^) or incidentally (*H*^αα^) associated with the slowest transverse relaxation rate (highest intensity) are colored in green and blue, respectively (see text) The outer ^1^H transitions, *H*^αβ^ and *H*^βα^, are shown in black and brown, respectively

Figure 1A shows the energy level diagram of an isolated AMX (^15^N^1^H_1_^1^H_2_) spin system (NH_2_ group). Each eigenstate is defined by three symbols, ‘α’ or ‘β’, where the first symbol corresponds to the state of the ^15^N spin, and the last two symbols, to the spin states of the two hydrogens, H_1_ and H_2_. The 2D multiplet pattern observed for an individual ^1^H spin of an ^15^N^1^H_1_^1^H_2_ spin-system in a fully coupled HSQC experiment, is schematically shown in Fig. 1B, with the differences in peak widths intentionally exaggerated for illustration purposes. Among the four ^15^N transitions, the two central ones (*N*^αβ^ and *N*^βα^) are not observable in the limit where the one-bond ^15^N–H *J* couplings, ^1^*J*_NH1_ and ^1^*J*_NH2_, are equal. Although differences in ^1^*J*_NH_ of up to ~3 Hz have been reported for NH_2_ groups in model compounds (Bystrov 1976; McIntosh et al. 1997) — ^1^*J*_NH_ = − 93.2 ± 1.3 Hz between ^15^N and the *E* proton (*anti* to carbonyl oxygen) and ^1^*J*_NH_ = − 90.2 ± 0.9 Hz between ^15^N and the *Z* proton (*syn* to carbonyl oxygen) — these differences are predicted to be on a par with the linewidths of the central ^15^N transitions only for small proteins. In the ^1^H (horizontal) dimension, the multiplet pattern consists of a doublet of doublets, with the geminal two-bond ^1^H–^1^H *J* couplings, ^2^*J*_HH_, in NH_2_ groups of carboxamides small and *positive* (+ 2.3 ± 0.2 Hz in formamide (Chuck et al. 1969) and + 2.9 ± 0.5 Hz measured for 8 Asn/Gln NH_2_ groups in ubiquitin (Permi et al. 1999)). As ^2^*J*_HH_ is in practice always much smaller than the difference in chemical shifts between the protons *E* and *Z*, no strong coupling effects are expected for NH_2_ groups under any circumstances. Below we formulate the theoretical basis for differential relaxation of ^15^N and ^1^H transitions in an ^15^N–^1^H_2_ spin-system and identify ^15^N and ^1^H transitions associated with the slowest transverse relaxation rates (shown in red and green/blue in Fig. 1 for ^15^N and ^1^H, respectively).

Each of the four ^15^N transitions of an NH_2_ spin-system (Fig. 1A) can be described by a single-transition operator, *N*^*PQ*^, representing a combination of product operator terms (Sorensen et al. 1983),

1 $$N^{PQ} = N_{ \pm } + p \times 2N_{ \pm } H_{Z,1} + q \times 2N_{ \pm } H_{Z,2} + pq \times 4N_{ \pm } H_{Z,1} H_{Z,2}$$

where *P* and *Q* denote the eigenstates of the two protons *H*_Z,1_ and *H*_Z,2_, respectively: (*P*,*Q*) ∈ {(α,α); (α,β); (β,α); (β,β)}; *H*_Z_ = (*H*^α^–*H*^β^)/2; and the multiplicative factors *p* and *q* are equal to + 1 and -1 for (*P*,*Q*) in the α and β states, respectively: (*p*,*q*) ∈ {(1, 1); (1,− 1); (− 1, 1); (− 1,− 1)} (see Supplementary Information, SI, for expansion of Eq. 1 into the vector $\vec{L}$ of all 4 ^15^N components, Eq. S1). The cumulative rate of decay of transverse ^15^N magnetization in an isolated ^15^N–^1^H_2_ spin-system, *R*(*N*^*PQ*^), is composed of the following combination of transverse relaxation rates,

2 $$R(N^{PQ} ) = R_{{{\text{NH}}_{1} }}^{{{\text{DD}}}} + R_{{{\text{NH}}_{2} }}^{{{\text{DD}}}} + R_{{\text{N}}}^{{{\text{CSA}}}} + pq \times R_{{{\text{NH}}_{1} {\text{,NH}}_{2} }}^{{\text{DD,DD}}} + p \times R_{{{\text{N,NH}}_{1} }}^{{\text{CSA,DD}}} + q \times R_{{{\text{N,NH}}_{2} }}^{{\text{CSA,DD}}}$$

where $R_{{\text{A(M)}}}^{j}$ and $R_{{\text{A(M),AX}}}^{j,k}$ are auto-and cross-correlated relaxation matrices for nuclei A(M), respectively, due to relaxation mechanisms *j*, *k* ∈ {CSA, DD}. The first two terms in Eq. 2 ($R_{{{\text{NH}}_{1} }}^{{{\text{DD}}}} ,_{{}} R_{{{\text{NH}}_{2} }}^{{{\text{DD}}}}$) account for relaxation due to dipolar interactions of ^15^N with the two ^1^H spins; the third term ($R_{{\text{N}}}^{{{\text{CSA}}}}$) describes relaxation due to ^15^N CSA; the fourth term ($R_{{{\text{NH}}_{1} {\text{,NH}}_{2} }}^{{\text{DD,DD}}}$) accounts for cross-correlated relaxation between the two dipoles (N–H_1_ and N–H_2_); and the last two terms ($R_{{{\text{N,NH}}_{1} }}^{{\text{CSA,DD}}} ,R_{{{\text{N,NH}}_{2} }}^{{\text{CSA,DD}}}$) account for cross-correlated relaxation between ^15^N CSA and the two N–H dipoles. In the macromolecular limit (with only the contributions from the spectral density function *J* at zero frequency, *J*(0), taken into account), and assuming an axially symmetric ^15^N CSA tensor, these rates are given by,

3a $$R_{{{\text{NH}}_{1} }}^{{{\text{DD}}}} = R_{{{\text{NH}}_{2} }}^{{{\text{DD}}}} = \frac{1}{5}\left( {k_{{{\text{NH}}}}^{{{\text{DD}}}} } \right)^{2} S^{2} \tau_{C}$$

3b $$R_{{\text{N}}}^{{{\text{CSA}}}} = \frac{4}{45}\left( {k_{{\text{N}}}^{{{\text{CSA}}}} } \right)^{2} S^{2} \tau_{C}$$

3c $$R_{{NH_{1} NH_{2} }}^{DD,DD} = \frac{2}{5}\left( {k_{{{\text{NH}}}}^{{{\text{DD}}}} } \right)^{2} P_{2} \left( {\cos \theta_{{{\text{NH}}_{1} {\text{,NH}}_{2} }}^{{\text{DD,DD}}} } \right)S^{2} \tau_{C}$$

3d $$R_{{{\text{N,NH}}_{1} }}^{{\text{CSA,DD}}} = \frac{4}{15}k_{{\text{N}}}^{{{\text{CSA}}}} k_{{{\text{NH}}}}^{{{\text{DD}}}} P_{2} \left( {\cos \theta_{{{\text{N,NH}}_{1} }}^{{\text{CSA,DD}}} } \right)S^{2} \tau_{C}$$

3e $$R_{{{\text{N,NH}}_{2} }}^{{\text{CSA,DD}}} = \frac{4}{15}k_{{\text{N}}}^{{{\text{CSA}}}} k_{{{\text{NH}}}}^{{{\text{DD}}}} S^{2} \tau_{C} P_{2} \left( {\cos \theta_{{{\text{N,NH}}_{2} }}^{{\text{CSA,DD}}} } \right)S^{2} \tau_{C}$$

where $k_{NH}^{DD} = - \left( {\mu_{0} /4\pi } \right)\hbar \gamma_{N}^{{}} \gamma_{H}^{{}} r_{NH}^{ - 3}$; $k_{{\text{N}}}^{{{\text{CSA}}}} = \gamma_{{\text{N}}}^{{}} \Delta \sigma_{{\text{N}}} B_{0}$; μ_0_ is the vacuum permeability constant; *γ*_*i*_, the gyromagnetic ratio of spin *i*; *r*_NH_, the N–H inter-nuclear distance (1.02 Å); *τ*_*C*_, the global molecular rotational correlation time (assumed isotropic); *S*, the generalized order parameter (assumed the same for all types of interactions); Δσ_N_, ^15^N CSA, Δσ = σ_*ZZ*_–(σ_*XX*_ + σ_*YY*_)/2 (see ‘Materials and Methods’); *B*_0_, the static magnetic field; and the second-order Legendre polynomial, *P*_2_(cos(*θ*_*μ*,*ν*_)) = (1/2)[3cos^2^(*θ*_*μ*,*ν*_)–1], where *θ*_*μ*,*ν*_ is the angle formed between the principal (*ZZ*) axes of tensorial interactions *μ* and *ν*. The angle $\theta_{{{\text{NH}}_{1} {\text{,NH}}_{2} }}^{{\text{DD,DD}}}$ = 118º was used in all calculations. Equation 2 does not include contributions to relaxation from ^1^H–^1^H dipolar interactions within NH_2_ groups (affecting only the central transitions of the NH_2_ multiplet) as well as dipolar interactions with ^1^H spins in the environment (protons ‘external’ to the NH_2_ group in question). The full treatment is provided in the SI, Eqs. S2–S3.

The four transitions of each ^1^H spin (Fig. 1A; H_1_ is singled out below) are described by,

4 $$H^{PQ} = H_{ \pm ,1} - p \times 2H_{ \pm ,1} N_{Z} + q \times 2H_{ \pm ,1} H_{Z,2} - pq \times 4H_{ \pm ,1} N_{Z} H_{Z,2}$$

where *P* and *Q* describe the eigenstates of ^15^N and H_2_, respectively; *N*_Z_ is defined as (*N*^β^–*N*^α^)/2; and the same convention for (*P*,*Q*) and (*p*,*q*) is used as in Eq. 1 (see SI for expansion of Eq. 4 into the vector $\vec{L}_{{\text{H}}}$ of all 4 ^1^H components, Eq. S4). The cumulative rate of decay of transverse ^1^H magnetization in an isolated NH_2_ group, *R*(*H*^*PQ*^), is comprised of the following contributions,

5 $$R(H^{PQ} ) = R_{{{\text{HN}}}}^{{{\text{DD}}}} + R_{{{\text{HH}}}}^{{{\text{DD}}}} + R_{{\text{H}}}^{{{\text{CSA}}}} + pq \times R_{{\text{HN,HH}}}^{{\text{DD,DD}}} + p \times R_{{\text{H,HN}}}^{{\text{CSA,DD}}} + q \times R_{{\text{H,HH}}}^{{\text{CSA,DD}}}$$

The first two terms in Eq. 5, $R_{{{\text{HN}}}}^{{{\text{DD}}}}$ and $R_{{{\text{HH}}}}^{{{\text{DD}}}}$, describe auto-relaxation due to dipolar interactions of a given ^1^H spin with ^15^N and another ^1^H spin, respectively; the third term ($R_{{\text{H}}}^{{{\text{CSA}}}}$) accounts for auto-relaxation due to ^1^H CSA; the fourth term ($R_{{\text{HN,HH}}}^{{\text{DD,DD}}}$) accounts for cross-correlated relaxation between the H–N and H–H dipoles; and the last two terms ($R_{{\text{H,HN}}}^{{\text{CSA,DD}}}$, $R_{{\text{H,HH}}}^{{\text{CSA,DD}}}$) account for cross-correlated relaxation between ^1^H CSA and H–N or H–H dipoles, respectively. In the macromolecular limit, and assuming an axially symmetric ^1^H CSA tensor, these rates are given by,

6a $$R_{{{\text{HN}}}}^{{{\text{DD}}}} = \frac{1}{5}\left( {k_{{{\text{HN}}}}^{{{\text{DD}}}} } \right)^{2} S^{2} \tau_{C}$$

6b $$R_{{{\text{HH}}}}^{{{\text{DD}}}} = \frac{1}{4}\left( {k_{{{\text{HH}}}}^{{{\text{DD}}}} } \right)^{2} S^{2} \tau_{C}$$

6c $$R_{{\text{H}}}^{{{\text{CSA}}}} = \frac{4}{45}\left( {k_{{\text{H}}}^{{{\text{CSA}}}} } \right)^{2} S^{2} \tau_{C}$$

6d $$R_{{\text{HN,HH}}}^{{\text{DD,DD}}} = \frac{2}{5}\left( {k_{{{\text{HN}}}}^{{{\text{DD}}}} } \right)\left( {k_{{{\text{HH}}}}^{{{\text{DD}}}} } \right)P_{2} \left( {\cos \theta_{{\text{HN,HH}}}^{{\text{DD,DD}}} } \right)S^{2} \tau_{C}$$

6e $$R_{{\text{H,HN}}}^{{\text{CSA,DD}}} = \frac{4}{15}k_{{\text{H}}}^{{{\text{CSA}}}} k_{{{\text{HN}}}}^{{{\text{DD}}}} P_{2} \left( {\cos \theta_{{\text{H,HN}}}^{{\text{CSA,DD}}} } \right)S^{2} \tau_{C}$$

6f $$R_{{\text{H,HH}}}^{{\text{CSA,DD}}} = \frac{4}{15}k_{{\text{H}}}^{{{\text{CSA}}}} k_{{{\text{HH}}}}^{{{\text{DD}}}} P_{2} \left( {\cos \theta_{{\text{H,HH}}}^{{\text{CSA,DD}}} } \right)S^{2} \tau_{C}$$

where in addition to the definitions for Eq. 3: $k_{HN}^{DD} = k_{NH}^{DD}$; $k_{HH}^{DD} = - \left( {\mu_{0} /4\pi } \right)\hbar \gamma_{{\text{H}}}^{2} r_{{{\text{HH}}}}^{ - 3}$; *r*_HH_ is the ^1^H–^1^H inter-nuclear distance (1.74 Å); $k_{{\text{H}}}^{{{\text{CSA}}}} = \gamma_{{\text{H}}}^{{}} \Delta \sigma_{{\text{H}}} B_{0}$, where Δσ_H_ is the ^1^H CSA. The angle $\theta_{{\text{HN,HH}}}^{{\text{DD,DD}}}$ = 31º was used in all calculations. Equation 5 does not include contributions to ^1^H relaxation from dipolar interactions with external ^1^H spins—see SI, Eqs. S5-S6, for the full treatment.

**Fig. 2:**
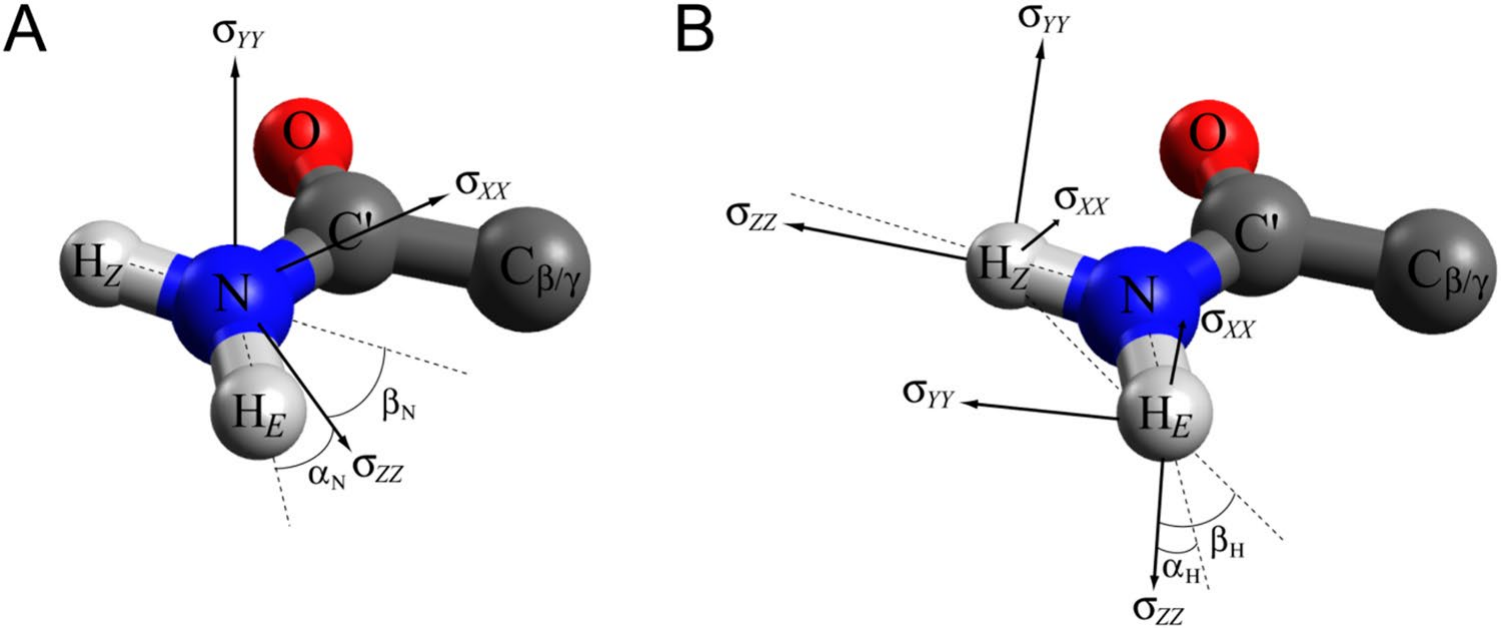
Typical orientations of the CSA tensors of (**A**) ^15^N nuclei, and (**B**) ^1^H_*Z*_ and ^1^H_*E*_ nuclei in carboxamide NH_2_ groups. The directions of N–H bond vectors and the vectors connecting the two ^1^H nuclei are extended with short dashed lines. The angles $\theta_{{{\text{N,NH}}_{1} }}^{{\text{CSA,DD}}}$ and $\theta_{{{\text{N,NH}}_{2} }}^{{\text{CSA,DD}}}$ in Eqs. 3d–e are labeled by ‘α_N_’ and ‘β_N_’, respectively, in (A). The angles $\theta_{{\text{H,HN}}}^{{\text{CSA,DD}}}$ and $\theta_{{\text{H,HH}}}^{{\text{CSA,DD}}}$ in Eqs. 6e–f are labeled by ‘α_H_’ and ‘β_H_’, respectively, in (B). See ‘Materials and Methods’ for details of DFT calculations and SI, Tables S1 and S2 for the principal values and orientations of the ^15^N and ^1^H_*E*_/^1^H_*Z*_ CSA tensors, respectively, obtained from the calculation with the program Gaussian for non-hydrogen-bonded and hydrogen-bonded side chain NH_2_ group of Gln41 in the protein ubiquitin

To gain quantitative insight into the magnitudes of ^15^N and ^1^H CSA (Δσ) and the angular terms in expressions for the CSA/DD cross correlated relaxation rates in Eqs. 3d–e and 6e–f, we performed DFT calculations of ^15^N and ^1^H CSA tensors in carboxamide NH_2_ groups (see ‘Materials and Methods’ and SI, Tables S1-S2). Figures 2A and B show typical orientations of ^15^N and ^1^H CSA tensors, respectively, relative to the carboxamide moiety. The calculated ^15^N Δσ is ~ −152 ppm, and is not significantly affected by protein environment (hydrogen bonding). The *ZZ* axis of the ^15^N CSA tensor lies practically (to within ~3º) in the carboxamide plane and forms an angle of ~20º with the bond vector connecting ^15^N with the *E* hydrogen, N-H_*E*_ (the angle $\theta_{{{\text{N,NH}}_{1} }}^{{\text{CSA,DD}}}$ in Eq. 3d, labeled as ‘α_N_’ in Fig. 2A), and an angle of ~ 140º (or 40º since *P*_2_[*cos*(*θ*)] = *P*_2_[*cos*(180º-*θ*)]) with the bond vector connecting ^15^N with the *Z* hydrogen, N–H_*Z*_ (the angle $\theta_{{{\text{N,NH}}_{2} }}^{{\text{CSA,DD}}}$ in Eq. 3e, labeled as ‘β_N_’ in Fig. 2A). The *XX* axis of the ^15^N CSA tensor is almost collinear (to within ~10–12º) with the N–C′ bond, while the *YY* axis is aligned with the normal to the carboxamide plane to within ~3–5º (Fig. 2A). These results (see typical values in the SI, Table S1) are in good agreement with earlier calculations of ^15^N CSA tensors in carboxamide NH_2_ groups of Asn/Gln side chains by Ernst and co-workers (Scheurer et al. 1999).

The calculated ^1^H CSA tensors are more sensitive to hydrogen bonding: the Δσ_H_ values of  ~8 and ~11 ppm for the *E* and *Z* hydrogens, respectively, were obtained in the absence of hydrogen bonding, while for the hydrogen-bonded cases, Δσ_H_ generally increases to ~12–15 ppm for both hydrogens (note that although the calculated ^1^H CSA tensors are usually significantly asymmetric, we assume axial symmetry for the purposes of this discussion—see SI for exact representation of ^1^H CSA used in all calculations of relaxation rates and Table S2 for typical ^1^H_*E/Z*_ CSA values). The *ZZ* axes of the CSA tensors of H_*E*_ and H_*Z*_ lie almost in the carboxamide plane (typically, within up to ~12º) forming the angles $\theta_{{\text{H,HN}}}^{{\text{CSA,DD}}}$ (labeled with ‘α_H_’ in Fig. 2B) of ~20 ± 12º with respect to their respective H-N bond vectors, while the angles between the *ZZ* axes and the H–H vectors ($\theta_{{\text{H,HH}}}^{{\text{CSA,DD}}}$; labeled with ‘β_H_’ in Fig. 2B) are larger, ~50 ± 12º. Note that from the geometry of the NH_2_ group, if the principal axis of the ^1^H CSA tensor was collinear with the N–H bond vector (*i.e.* $\theta_{{\text{H,HN}}}^{{\text{CSA,DD}}}$(α_H_) = 0), the angle $\theta_{{\text{H,HH}}}^{{\text{CSA,DD}}}$(β_H_) would be ~30º (Fig. 2B). It can also be noted from DFT calculations that both angles $\theta_{{\text{H,HN}}}^{{\text{CSA,DD}}}$(α_H_) and $\theta_{{\text{H,HH}}}^{{\text{CSA,DD}}}$(β_H_) tend to decrease by ~10–15º upon hydrogen bonding implying that hydrogen bonding reorients the *ZZ* axis of the ^1^H CSA tensor somewhat closer to the corresponding N–H bond vector.

**Fig. 3:**
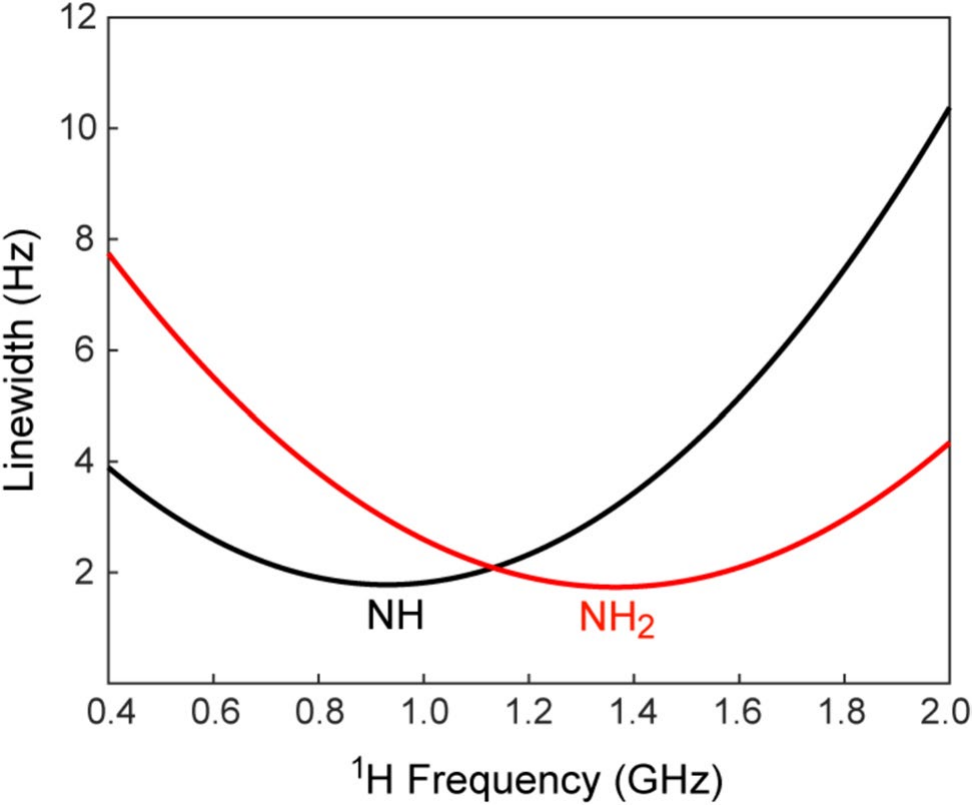
Plots showing the dependence of ^15^N TROSY linewidths (^15^N*-R*_2_/π; Hz) on the strength of the static magnetic field, *B*_0_, expressed in units of ^1^H frequency (GHz) for isolated ^15^N–^1^H (black) and ^15^N–^1^H_2_ (red) spin-systems. The calculations were performed for the product *S*^2^*τ*_*C*_ equal to 25 ns using the angles $\theta_{{{\text{N,NH}}_{1} }}^{{\text{CSA,DD}}}$ = 20º and $\theta_{{{\text{N,NH}}_{2} }}^{{\text{CSA,DD}}}$ = 40º for ^15^N–^1^H_2_ spin-systems, and the angle $\theta_{{\text{N,NH}}}^{{\text{CSA,DD}}}$ = 16º for ^15^N–^1^H spin-systems. ^15^N CSA (Δσ_N_) values of  -152 ppm and -164 ppm for NH_2_ and NH groups, respectively, were used, along with the N–H internuclear distance *r*_NH_ = 1.02 Å for both spin-systems. Note that the minimal ^15^N linewidths of NH_2_ groups (red) is ever so slightly narrower (by 2.5%) than that of their NH counterparts (black). This is a consequence of NH-NH dipole–dipole cross correlated relaxation in NH_2_ spin-systems, Eq. (3c). Although relatively small in magnitude (see text), these cross correlations lead to the same narrowing of the lines of both the TROSY (*N*^ββ^) and anti-TROSY (*N*^αα^) ^15^N components in NH_2_ groups. When the contribution of NH–NH dipole–dipole cross correlated relaxation is excluded from the calculation, the minimal linewidth for NH_2_ groups becomes 2.6-fold broader than that of its NH counterpart (the red curve shifting upwards by ~2.7 Hz)

Calculations of ^15^N relaxation rates in NH_2_ groups using Eqs. 2, 3 (see also SI, Eqs. S2-S3) show that NH_1_–NH_2_ dipole–dipole cross-correlated relaxation ($R_{{{\text{NH}}_{1} {\text{,NH}}_{2} }}^{{\text{DD,DD}}}$, Eq. 3c) does not play a major role in ^15^N line narrowing, as the angle $\theta_{{{\text{NH}}_{1} {\text{,NH}}_{2} }}^{{\text{DD,DD}}}$ is ~118º (*P*_2_[cos(118º)] = − 0.17), and the contributions of this cross-correlated relaxation mechanism are the same for the *N*^αα^ and *N*^ββ^ transitions. The physical origin of line narrowing for the *N*^ββ^ transitions (shown in red in Fig. 1) derives from the cross-correlated relaxation between ^15^N CSA and the two N–H dipoles ($R_{{{\text{N,NH}}_{1} }}^{{\text{CSA,DD}}}$ and $R_{{{\text{N,NH}}_{2} }}^{{\text{CSA,DD}}}$, Eqs. 3d–e), as is the case for ^15^N-^1^H spin-systems (Pervushin et al. 1997; Goldman 1984). However, the line-narrowing effects induced by the CSA/DD cross-correlated relaxation are substantially weaker in ^15^N–^1^H_2_ spin-systems compared to their ^15^N-^1^H counterparts for the simple reason that ^15^N nuclei are relaxed by dipolar interactions with two ^1^H spins (Eqs. 2 and 3a), while the *ZZ* axis of the ^15^N CSA tensor is approximately aligned only with the N-H_*E*_ bond vector (angle α_N_ in Fig. 2A; see discussion above and (Scheurer et al. 1999)). As a consequence, the CSA/DD cross correlated relaxation rates in Eqs. 3d–e differ by a factor of ~2 (for $\theta_{{{\text{N,NH}}_{1} }}^{{\text{CSA,DD}}}$(α_N_) = 20º and $\theta_{{{\text{N,NH}}_{2} }}^{{\text{CSA,DD}}}$(β_N_) = 40º, *P*_2_(α_N_)/*P*_2_(β_N_) = 2.2), and the relaxation due to the N-H_*Z*_ dipolar interaction is not compensated for by the CSA/DD cross correlation to the same extent as that due to the N-H_*E*_ dipole–dipole interaction. The field dependence of ^15^N TROSY linewidths for ^15^N–^1^H and ^15^N–^1^H_2_ spin-systems are compared in Fig. 3, showing that the optimal ^15^N TROSY effect for 15N–1H2 spin-systems is achieved at ~40% higher magnetic field strength than for their 15N–1H counterparts.

The effects of cross-correlated relaxation on the relative relaxation rates/intensities of the ^1^H transitions in ^15^N–^1^H_2_ spin-systems are more subtle. Unlike in ^15^N–^1^H spin-systems, the cross-correlated relaxation between the H–N and H–H dipoles ($R_{{\text{HN,HH}}}^{{\text{DD,DD}}}$; Eq. 6d) is the main determinant of line narrowing of the *H*^αα^ and *H*^ββ^ transitions in NH_2_ groups—the inner lines of the NH_2_ quadruplet, shown in blue and green, respectively, in Fig. 1. With the angle $\theta_{{\text{HN,HH}}}^{{\text{DD,DD}}}$ = 30º, these cross-correlated relaxation rates have the same negative contributions to the rates of the *H*^αα^ and *H*^ββ^ transitions (Eq. 5), that amount to ~1/2 of the auto-relaxation rates due to H–H dipolar interactions ($R_{{{\text{HH}}}}^{{{\text{DD}}}}$). The relative relaxation rates/intensities of the *H*^αα^ and *H*^ββ^ transitions are dependent on the interplay between the ^1^H CSA/HN DD and ^1^H CSA/HH DD cross-correlated relaxation mechanisms ($R_{{\text{H,HN}}}^{{\text{CSA,DD}}}$ and $R_{{\text{H,HH}}}^{{\text{CSA,DD}}}$, Eqs. 6e–f). The calculated angles $\theta_{{\text{H,HN}}}^{{\text{CSA,DD}}}$ ~20 ± 12º and $\theta_{{\text{H,HH}}}^{{\text{CSA,DD}}}$ ~50 ± 12º (the angles α_H_ and β_H_, respectively, in Fig. 2B), lead to the following relationships: ($R_{{\text{H,HN}}}^{{\text{CSA,DD}}}$ < 0; $R_{{\text{H,HH}}}^{{\text{CSA,DD}}}$ > 0; |$R_{{\text{H,HN}}}^{{\text{CSA,DD}}}$| >|$R_{{\text{H,HH}}}^{{\text{CSA,DD}}}$|) for the *H*^ββ^ transitions, and ($R_{{\text{H,HN}}}^{{\text{CSA,DD}}}$ > 0; $R_{{\text{H,HH}}}^{{\text{CSA,DD}}}$ < 0; |$R_{{\text{H,HN}}}^{{\text{CSA,DD}}}$| >|$R_{{\text{H,HH}}}^{{\text{CSA,DD}}}$|) for the *H*^αα^ transitions. As a consequence, in the vast majority of NH_2_^1^H multiplets inspected, the rates $R_{{\text{H,HN}}}^{{\text{CSA,DD}}}$ dominate, leading to the narrowest (most intense) lines in the ^1^H dimension of 2D NMR spectra corresponding to the *H*^ββ^ transitions (green in Fig. 1). However, a small number of exceptions when the transitions *H*^αα^ (blue in Fig. 1) and *H*^ββ^ have the same intensities or the former is slightly higher, were noted in the NMR spectra, and can be associated with those cases where $\theta_{{\text{H,HN}}}^{{\text{CSA,DD}}}$(α_H_) <  ~12º, $\theta_{{\text{H,HH}}}^{{\text{CSA,DD}}}$(β_H_) <  ~ 38º, and |$R_{{\text{H,HH}}}^{{\text{CSA,DD}}}$| >|$R_{{\text{H,HN}}}^{{\text{CSA,DD}}}$|. These rare instances may tentatively be attributed to strong hydrogen bonds formed by NH_2_ hydrogen(s) with oxygen acceptors in carbonyl groups of the protein or water molecules.

Hindered rotation about the C_γ_–N (Asn) and C_δ_–N_ε_ (Gln) bonds that interchange the two amide protons, will not affect the net results of the relaxation effects discussed here. Earlier NMR measurements performed at 35 ºC, yielded rotation rates in the range 0–1 s^−1^ for side chains buried in the protein core, and 1–10 s^−1^ for mobile side chains (Guenneugues et al. 1997). A more recent NMR study (Wang et al. 2021) showed, however, that the rotation rates are highly temperature dependent and decrease by ~3–~5-fold upon a temperature decrease from 35 to 15 ºC. Since TROSY effects are demonstrated here with experiments performed at 5 ºC, we do not expect that rotation rates on the order of 1–2 s^−1^ would influence any of the cross-correlated relaxation rates discussed above. We note that even in the case of anomalously high rotation rates at 5 ºC, the individual ^15^N CSA/N–H DD cross-correlated relaxation rates ($R_{{{\text{N,NH}}_{1} }}^{{\text{CSA,DD}}}$ and $R_{{{\text{N,NH}}_{2} }}^{{\text{CSA,DD}}}$ in Eqs. 3d–e) will be partially averaged without net effects on the relaxation rates of the *N*^αα^ or *N*^ββ^ transitions. Likewise, as the principal values and orientations of the ^1^H CSA tensors for the protons *E* and *Z* are similar (see ‘Materials and Methods’), the bond rotation that inter-changes these two protons is not expected to significantly affect the relaxation rates of each individual ^1^H spin.

### Experimental verification

**Fig. 4:**
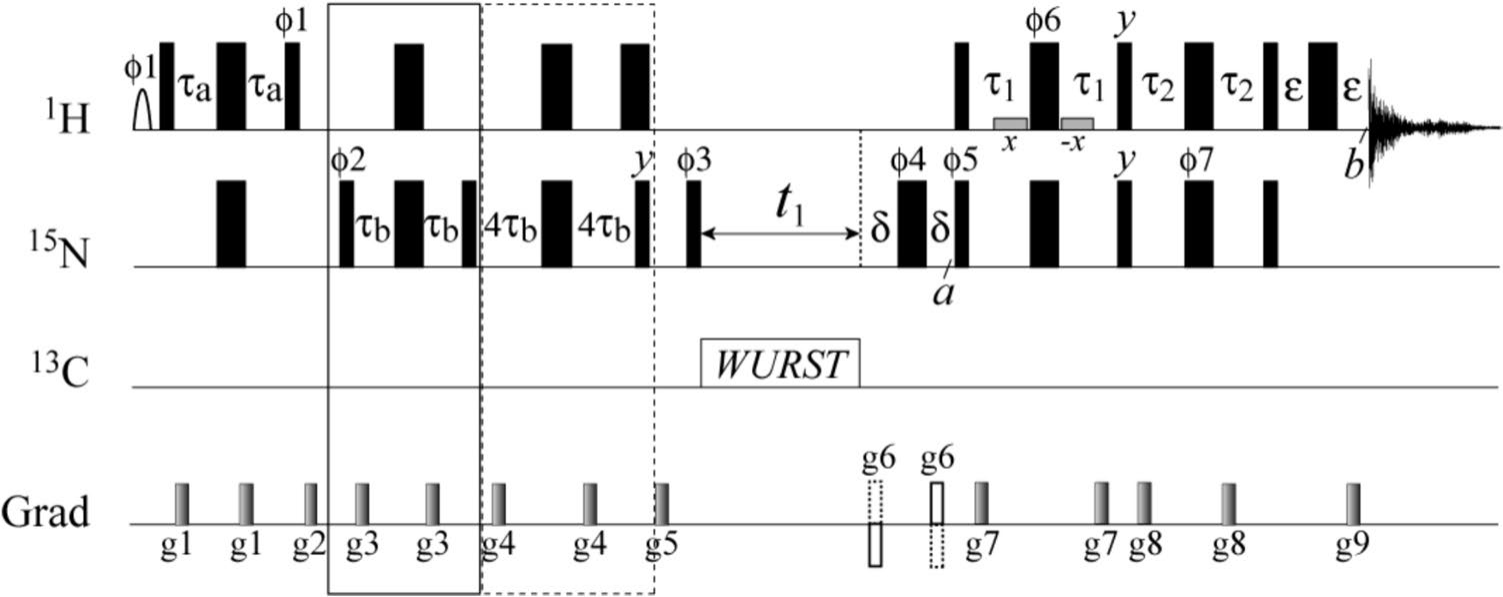
Pulse scheme for selection of each of the eight NH_2_ multiplet components. All narrow and wide rectangular pulses are applied with flip-angles of 90° and 180°, respectively, along the *x*-axis unless indicated otherwise. The ^1^H carrier is positioned at the water resonance, while the ^15^N carrier is placed at 110 ppm. All ^15^N pulses are applied with the highest possible power (RF field strength 6.25 kHz). The steady-state Boltzmann ^15^N polarization is added to the magnetization transferred from ^1^H by appropriate choice of the phase *ϕ*1. ^13^C decoupling is intended for U-[^13^C; ^15^N]-labeled samples and is achieved using 180º adiabatic WURST-20 pulses (Kupce and Freeman 1996) (2.5 ms; ± 30 kHz inversion bandwidth) in a p5m4 composite decoupling scheme (Tycko et al. 1985), with the ^13^C carrier placed at 110 ppm. These pulses are designed for simultaneous decoupling of carbonyl (^13^C′) and aliphatic (^13^C_α/β/γ_) carbon regions. The shaped ^1^H_ϕ1_ pulse at the beginning of the scheme is a ~7 ms 90º water selective pulse applied with an EBURP-1 profile (Geen and Freeman 1991). To achieve good suppression of the water signal for any phase of the ^1^H_ϕ6_ pulse (2τ_1_ period), this pulse is flanked by two rectangular water-selective ^1^H pulses (shown with shaded rectangles) of ~1.5 ms duration applied with the phases *x* and -*x*, respectively. Delays are as follows: τ_a_ = 2.25 ms; τ_b_ = 1/(16*J*_NH_) = 690 μs; τ_1_ = 0.34/(2*J*_NH_) = 1.89 ms; τ_2_ = 0.23/(2*J*_NH_) = 1.28 ms; δ = 800 μs; ε = 500 μs. The phase cycling for ϕ1, ϕ2, ϕ6 and ϕ7 is as indicated in Table 1; ϕ3 = 2(*x*), 2(*-x*); ϕ4 = 2(*x*), 2(*-x*), 2(*y*), 2(*-y*); ϕ5 = *x*; receiver = *x*,*-x*,-*x*,*x*,* -x*,*x*,*x*,*-x*. The durations and strengths of the pulsed-field gradients applied along the *z*-axis in units of (ms; G/cm) are: g1 = (0.4; 35), g2 = (1.5; 40), g3 = (0.3; 35), g4 = (0.5; 35), g5 = (1.0; 35), g6 = (0.5; 30), g7 = (0.1; 35), g8 = (0.4; 35), g9 = (0.102; 30). In addition, each of the encoding and decoding gradients g6 and g9 are applied along the *x* and *y* axes with the strengths of ~15 G/cm. Quadrature detection in *t*_1_ is achieved using the Rance-Kay gradient selection scheme (Kay et al. 1992; Schleucher et al. 1993), with ϕ5 inverted together with the gradients g6 for each complex point in *t*_1_

The pulse scheme for selection of each of the 8 NH_2_ multiplet components is shown in Fig. 4, and is based on the experiment developed for selection of ^1^H–^13^C multiplet components in ^13^CH_2_ groups by (Miclet et al. 2004). Table 1 lists the phase settings in the pulse scheme of Fig. 4 used for selection of each multiplet component. Following the transfer of magnetization to ^15^N, the element of duration 2τ_b_ (enclosed in a solid rectangle in Fig. 4) selects for the desired ^15^N component, with the phase ϕ2 controlling which component is isolated (*N*^αα^ or *N*^ββ^). Subsequently, the element of duration 8τ_b_ (enclosed in the dashed rectangle, Fig. 4) eliminates the signals from backbone N–H groups some of which would otherwise overlap with the Asn and Gln side chain NH_2_ correlations. Following the ZZ-filter (gradient g5), the chemical shift of the selected ^15^N component is evolved during the *t*_1_ acquisition period, and ^15^N magnetization is subsequently selectively transferred to one of the 4 ^1^H components of the H_*E*_ and H_*Z*_ protons by a variation of the planar Total Correlation Spectroscopy (TOCSY) element (Schulte-Herbrüggen et al. 1991; Mádi et al. 1997). This planar TOCSY based transfer can be distinguished from the SPITZE-HSQC approach developed earlier for simultaneous measurements of ^1^*D*_CH_ and ^1^*D*_HH_ residual dipolar couplings in ^13^CH_2_ groups of proteins (Carlomagno et al. 2000).

**Table 1:** Phase Settings in the Pulse Scheme of Fig. 4 for Selection of Each of the NH_2_ Multiplet Components^*a*)^

^15^N Transition	^1^H Transition	ϕ1	ϕ2	ϕ6	ϕ7
*N*^ββ^	*H*^ββ^	***− y***	**45º/225º**	***x***	***x***
	*H*^βα^	*− y*	45º/225º	*y*	*y*
	*H*^αβ^	*− y*	45º/225º	*x*	*y*
	*H*^αα^	*− y*	45º/225º	*y*	*x*
*N*^αα^	*H*^ββ^	*y*	135º/315º	*y*	*x*
	*H*^βα^	*y*	135º/315º	*x*	*y*
	*H*^αβ^	*y*	135º/315º	*y*	*y*
	*H*^αα^	*y*	135º/315º	*x*	*x*

^*a*^The phases for selection of the narrowest component are shown in bold

 The phases of 180º ^1^H pulses ϕ6 and ϕ7 (see Table 1) control which ^1^H component is selected as described in detail by (Miclet et al. 2004). Briefly, when the component *N*^ββ^ is isolated before the *t*_1_ period and it is assumed that ^1^*J*$_{{{\text{NH}}_{1} }}$ = ^1^*J*$_{{{\text{NH}}_{2} }}$ = ^1^*J*_NH_, between the time-points *a* and *b* of the pulse scheme in Fig. 4 with (ϕ6, ϕ7) = (*x*,* x*), the ^15^N magnetization is transferred to ^1^H according to the following transformation,

7 $$\begin{gathered} - N_{ \pm }^{\beta \beta } \mathop{\longrightarrow}\limits^{a - b}(H_{ \pm ,1}^{{}} - 4H_{ \pm ,1}^{{}} H_{Z,2}^{{}} N_{Z} )\left\{ {\sin (\pi J_{NH} \tau_{1} ) + \cos (\pi J_{NH} \tau_{1} )\sin (\pi J_{NH} \tau_{2} )} \right\} + \hfill \\ (2H_{ \pm ,1}^{{}} N_{Z} - 2H_{ \pm ,1}^{{}} H_{Z,2}^{{}} )\left\{ \begin{gathered} \cos (\pi J_{NH} \tau_{1} )\sin (\pi J_{NH} \tau_{1} )\cos (\pi J_{NH} \tau_{2} ) + \hfill \\ [1 + \sin^{2} (\pi J_{NH} \tau_{1} )]\cos (\pi J_{NH} \tau_{2} )\sin (\pi J_{NH} \tau_{2} ) \hfill \\ \end{gathered} \right\} \hfill \\ \end{gathered}$$

Durations of the delays τ_1_ and τ_2_ are found by equating the trigonometric terms in the curly brackets in Eq. 7 and maximizing their sum, to yield: τ_1_ = 0.34/(2*J*_NH_); τ_2_ = 0.23/(2*J*_NH_). The optimal efficiency of the *N*^ββ^ → *H*^ββ^ transfer *f* = 0.85 (Miclet et al. 2004), where 1 is the efficiency of the ^15^N → ^1^H transfer in the (fully decoupled) sensitivity-enhanced HSQC experiment optimized for ^15^N-^1^H_2_ spin-systems (Schleucher et al. 1994). It is important to note that the expression for the transformation in Eq. 7 holds only in the basis of spherical operators for ^15^N and ^1^H magnetization underscoring the importance of using pulsed field gradients for coherence selection.

The experiment in Fig. 4 was tested on the U-[^15^N]-labeled sample of the buried cavity mutant, L99A, of T4 lysozyme at 5 ºC (*τ*_*C*_ ~ 20 ns). Keeping in mind that comparison of sensitivities of the TROSY and decoupled HSQC spectra recorded with identical sampling (acquisition) times may be biased in favor of TROSY experiments (Miclet et al. 2004), we measured first the relaxation times of the ^1^H-decoupled ^15^N SQ coherences and the TROSY ^15^N component (*N*^ββ^) for 26 separated NH_2_ correlations of the T4 lysozyme mutant (5 ºC; 900 MHz). The average relaxation times, < T_2_ > , are 55 ± 19 ms and 130 ± 55 ms for the decoupled ^15^N coherences and *N*^ββ^ components, respectively. This helped us to choose an experimentally realistic scenario where the maximal acquisition time in the indirect (^15^N) dimension, *t*_1,max_, is set to a value approximately ‘half-way’ between these two averages (*t*_1,max_ = 88 ms was used in practice). This *t*_1_ acquisition time is on the one hand sufficient for resolving all NH_2_ correlations in the 2D spectra of the T4 lysozyme mutant, and on the other hand, is not excessively long to significantly bias the comparison of the two experiments.

**Fig. 5:**
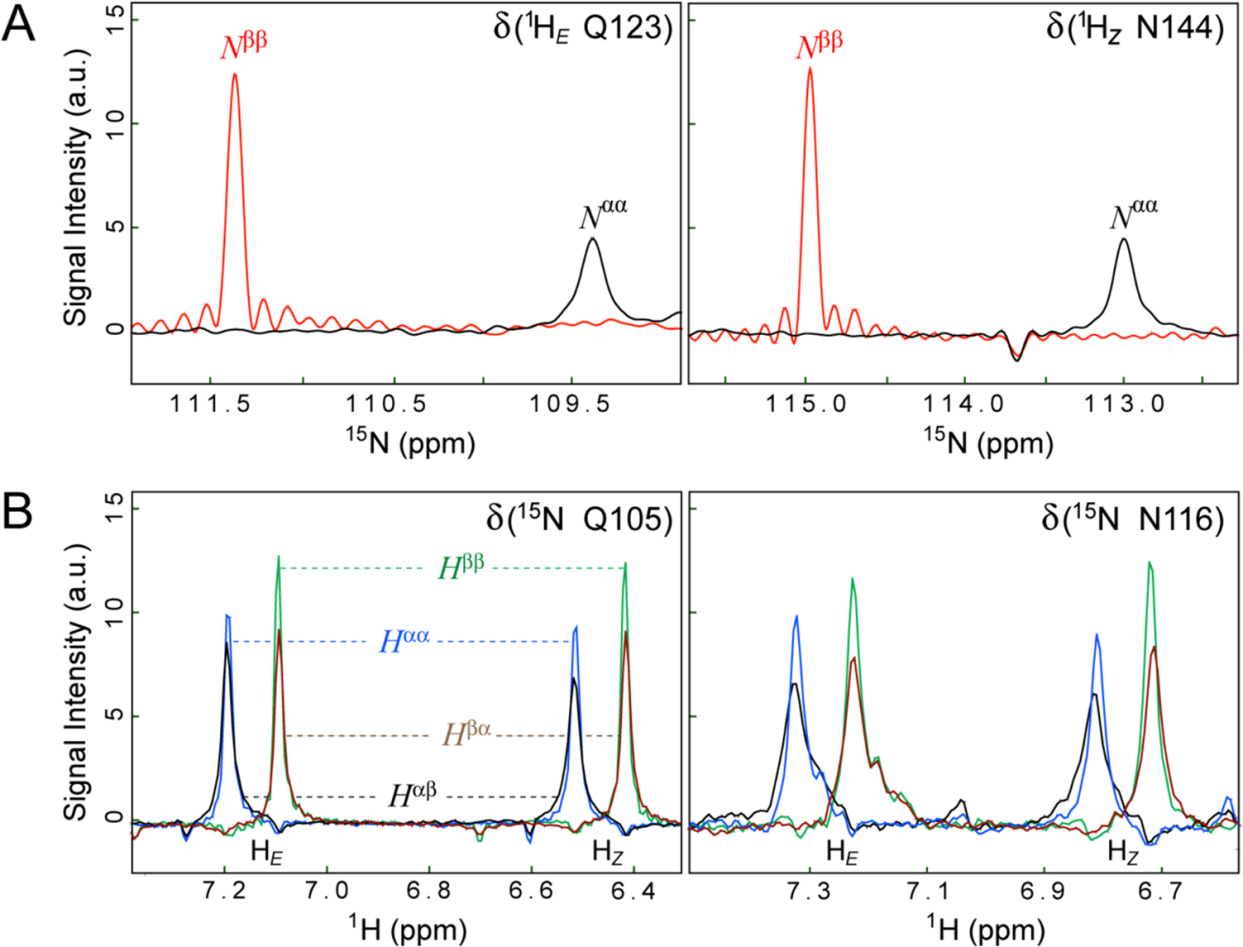
The components of the NH_2_ multiplet obtained for the buried cavity mutant of T4 lysozyme (5 ºC; 900 MHz) with the pulse scheme of Fig. 4. (**A**) 1D traces showing NH_2_ multiplet components along the ^15^N(*F*_1_) dimension of the 2D spectra recorded with the pulse scheme of Fig. 4 and selection of the narrowest ^1^H transitions (H^*ββ*^). The traces are drawn at the ^1^H chemical shifts δ of Gln123 and Asn144 of the T4 lysozyme mutant and are extracted from the 2D spectra processed without apodization in the ^15^N dimension. (**B**) 1D traces showing NH_2_ multiplet components along the ^1^H(*F*_2_) dimension of the 2D spectra acquired using the experiment of Fig. 4 with selection of the narrowest ^15^N transitions (N^*ββ*^). The traces are drawn at the ^15^N chemical shifts δ of Gln105 and Asn116, and are extracted from the 2D spectra processed without apodization in the ^1^H dimension. All multiplet components are colored with the same color-coding scheme as in Fig. 1

Selected components of the NH_2_ multiplet obtained with the pulse scheme of Fig. 4 for the T4 lysozyme mutant (5 ºC; 900 MHz; *t*_1, max_ = 88 ms) are shown in Fig. 5. The two multiplet components along the ^15^N dimension of the 2D spectra, corresponding to the ^15^N transitions *N*^αα^ and *N*^ββ^ (both obtained with selection of the narrowest ^1^H transitions, *H*^ββ^, in the ^1^H dimension of the 2D spectra), are shown in black and red, respectively, in Fig. 5A. Significant line narrowing is observed for the *N*^ββ^ components. While weaker than for ^15^N-^1^H spin-systems at the same *B*_0_ field (Fig. 3), this effect is strongly* B*_0_ field-dependent, as the physical origin of differential relaxation of the *N*^αα^ and *N*^*ββ*^ transitions derives from destructive interference of ^15^N CSA and N–H dipolar mechanisms (Eqs. 3e–d). The 4 multiplet components along the ^1^H dimension of the 2D spectra, corresponding to ^1^H transitions *H*^*PQ*^, (*P*,*Q*) ∈ {(α,α); (α,β); (β,α); (β,β)} (all obtained with selection of the narrowest ^15^N transitions, *N*^ββ^, in the ^15^N dimension of the 2D spectra), are shown for both *E* and *Z* protons in Fig. 5B. The differences in peak linewidths (intensities) are much less pronounced for ^1^H transitions, with the intensities *I* of components typically ordered as, *I*(*H*^ββ^) > *I*(*H*^αα^) > *I*(*H*^βα^) > *I*(*H*^αβ^), with rare exceptions when the order of the transitions *H*^ββ^ and *H*^αα^ is switched. As the cross correlated relaxation effects of the ^1^H CSA/H–N DD and ^1^H CSA/H–H DD varieties largely counteract each other, the H–N/H–H DD cross correlations constitute the dominant source of line narrowing in the ^1^H dimension (see ‘Theoretical Background’). In fact, the measurements of relative intensities of ^1^H components at different *B*_0_ field strengths (600, 800 and 900 MHz), showed that the line-narrowing effects in the ^1^H dimension are only weakly *B*_0_ field-dependent.

**Fig. 6:**
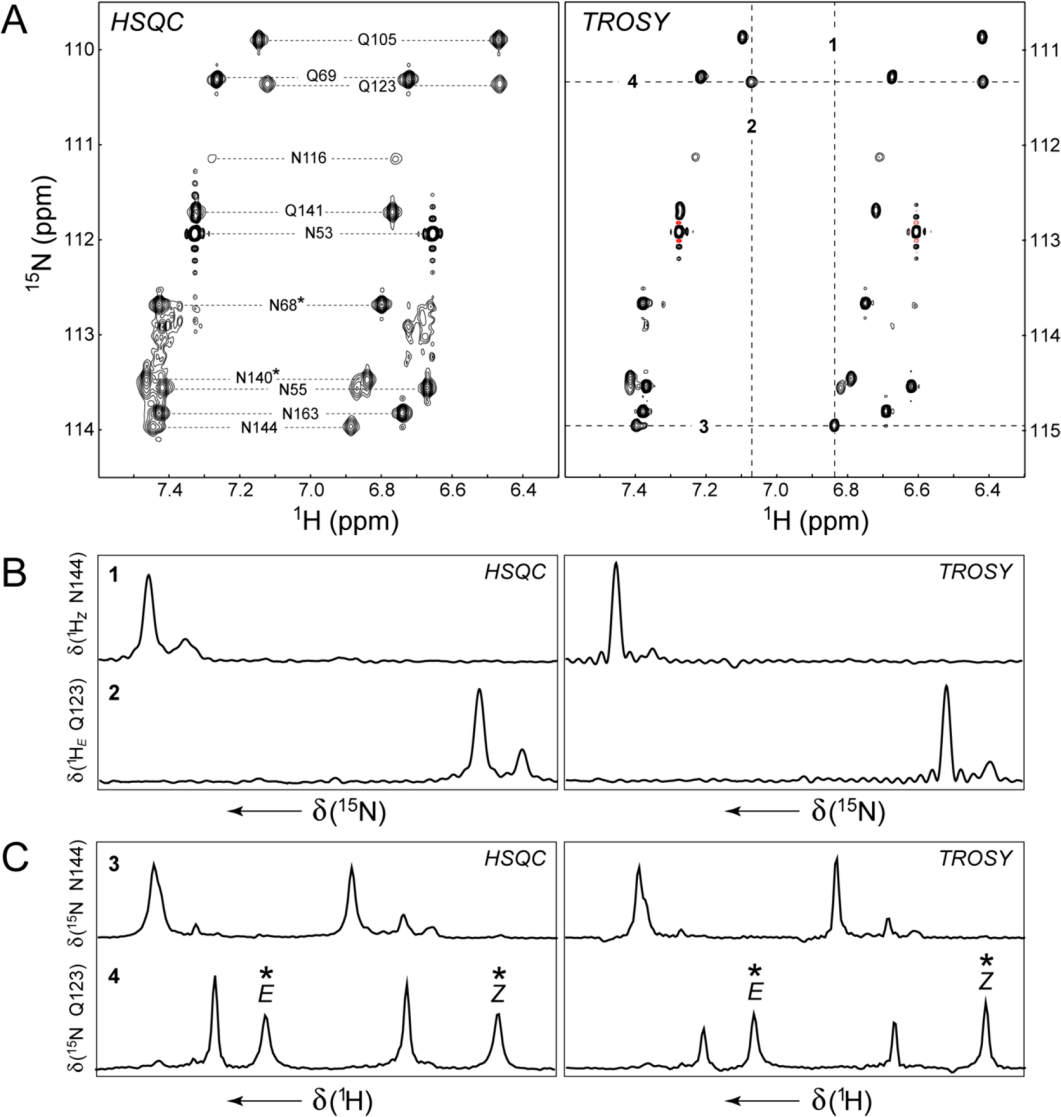
Comparison of a fully decoupled HSQC optimized for ^15^N–^1^H_2_ spin-systems with the NH_2_-TROSY experiment (the scheme of Fig. 4 with selection of the *N*^ββ^ and *H*^ββ^ components). **A** A region of 2D NH_2_ correlation maps obtained with the fully decoupled NH_2_-HSQC experiment (*Left panel*) and NH_2_-TROSY experiment (*Right panel*) recorded at 900 MHz on the U-[^15^N]-labeled sample of the buried cavity mutant of T4 lysozyme (5 ºC). The 2D spectra were processed using identical parameters, without apodization in either dimension, and plotted at the same contour levels. Note the different ^15^N chemical shift axes of the two panels chosen for easier comparison. Selected assignments of the NH_2_ sites are shown in the *Left panel*, with tentative assignments labeled with asterisks. **B** 1D traces taken along the ^15^N(*F*_1_) dimension of the 2D correlation maps at the frequencies δ marked by vertical long-dashed lines (labeled ‘**1’** and ‘**2**’ for the ^1^H_*Z*_ proton of Asn144 and ^1^H_*E*_ proton of Gln123, respectively) in the *Right panel* of (A). **C** 1D traces along the ^1^H(*F*_2_) dimension of the 2D correlation maps at the frequencies δ marked by horizontal long-dashed lines (labeled ‘**3**’ and ‘**4**’ for ^15^N of Asn144 and Gln123, respectively) in the *Right panel* of (A). The peaks reaching maximum intensity in the *Bottom row* (‘**4**’) are marked with asterisks

The comparison of 2D ^15^N–^1^H spectra obtained with a fully (^1^H/^15^N) decoupled gradient sensitivity enhanced HSQC optimized for NH_2_ groups (Schleucher et al. 1994) (performed without selection for *N*^ββ^ transitions but with the element selecting for NH_2_ groups of duration 8*τ*_b_ in Fig. 4 included) and the NH_2_-TROSY experiment (the pulse scheme of Fig. 4 with selection of the *N*^ββ^ and *H*^ββ^ components in the ^15^N and ^1^H dimensions, respectively, with ϕ1 = *-y*, ϕ2 = 45º/225º, ϕ6 = ϕ7 = *x*), for the T4 lysozyme L99A mutant (5 ºC; 900 MHz; *t*_1,max_ = 88 ms) is shown in Fig. 6A. Although on average the sensitivity (signal-to-noise ratio) of the NH_2_-TROSY spectrum is slightly lower (by ~12%) compared to its HSQC counterpart, a slight improvement in sensitivity is achieved for a third of the cross-peaks in the TROSY spectrum — likely those of more ordered Gln/Asn side chains. Considering that in the absence of relaxation, the sensitivity of the NH_2_-TROSY experiment is estimated to be a factor of 2/*f* ~2.4 lower than that of the NH_2_-HSQC, one can conclude that almost the same factor is ‘retrieved’ in NH_2_-TROSY owing to relaxation interference. Note that because of the high efficiency of the *N*^ββ^ → *H*^ββ^ transfer in the NH_2_-TROSY experiment, no sensitivity benefits are afforded by detection of *all* ^1^H components in combination with ^15^N decoupling during the *t*_2_ acquisition period.

The principal advantage of the NH_2_-TROSY experiment lies in improved resolution of the 2D correlation maps. Figures 6B and C show the 1D traces taken at selected positions along the ^15^N and ^1^H dimensions, respectively, of the 2D spectra. While reduction in linewidths is observed in the NH_2_-TROSY dataset in both dimensions, the line narrowing is more pronounced in the ^15^N dimension (Fig. 6B) due to efficient ^15^N CSA/N–H DD cross-correlated relaxation at high *B*_0_ field strengths. The relatively minor narrowing of lines in the ^1^H dimension of the NH_2_-TROSY spectra (Fig. 6C) is primarily the consequence of N–H/H–H DD/DD cross correlated relaxation effects. Note that, as opposed to aliphatic ^13^CH_2_ groups in proteins (Miclet et al. 2004), where |^2^*J*_HH_| is ~10–12 Hz, the narrowing of ^1^H lines in NH_2_-TROSY due to multiplet reduction/simplification is practically negligible, since ^2^*J*_HH_ couplings in NH_2_ groups are 4-to-5-fold smaller by absolute magnitude.

We estimate that typical contributions of dipolar interactions with external ^1^H spins to ^15^N and ^1^H auto-relaxation rates (see SI, Eqs. S2-S3 and S5-S6, for relaxation matrices $\tilde{\Gamma }_{{{\text{HH}}}}^{{{\text{ext}}}}$) in a protonated protein with *τ*_C_~20 ns, are on the order of 5 and 15 s^−1^, respectively. These contributions (in addition to conformational exchange experienced at some of the NH_2_ sites) can significantly ‘dilute’ the TROSY line-narrowing effects in both ^15^N and ^1^H dimensions of the NH_2_-TROSY spectra recorded on fully protonated protein samples, as is the case in the present work. Although some improvements in sensitivity and resolution of NH_2_-TROSY spectra may be expected upon protein deuteration (especially for hydrogen-bonded NH_2_ groups), these are predicted to be smaller than for backbone amide N–H groups, since generally faster exchange of NH_2_ protons with the water solvent will have similar, adverse effects on TROSY linewidths. Albeit not included in our treatment of relaxation in ^15^N–^1^H_2_ spin-systems here, exchange with water can be accounted for in a manner similar to the treatment of interactions with external ^1^H spins (Grzesiek and Bax 1993; Skrynnikov and Ernst 1999).

### Application to ^15^N CPMG relaxation dispersion in NH_2_ groups

**Fig. 7:**
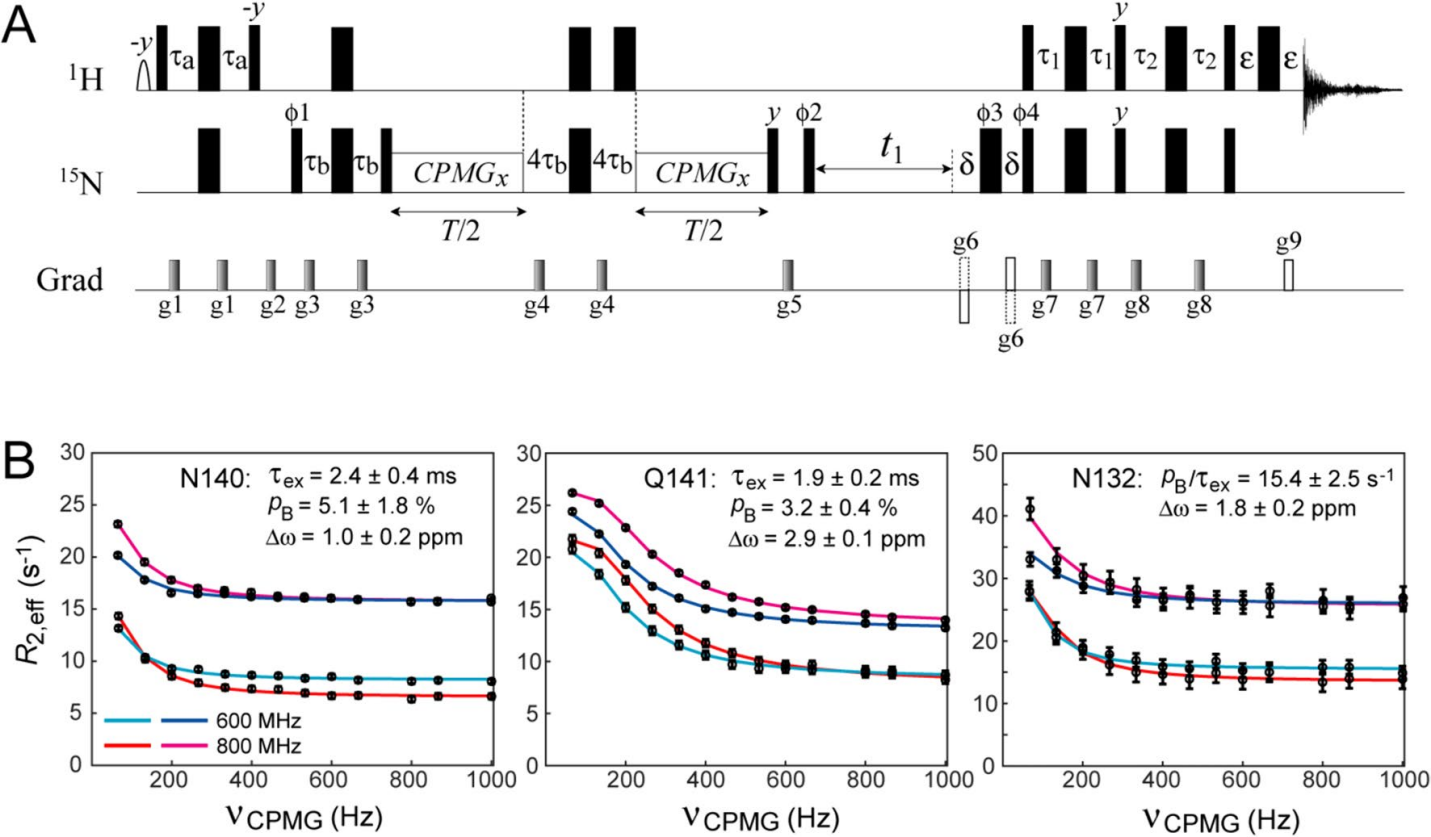
Application of NH_2_-TROSY to CPMG relaxation dispersion measurements. **A** Pulse scheme for the NH_2_-TROSY based ^15^N CPMG relaxation dispersion experiment. All experimental parameters including durations of delays and durations/strengths of pulsed field gradients, are as listed in Fig. 4. The delay *T* is the total duration of the relaxation period, during which the train of 180º ^15^N CPMG pulses is applied with phase *x*. The phase cycling is: ϕ1 = 45º, 225º; ϕ2 = 2(*x*), 2(*-x*); ϕ3 = 2(*x*), 2(*-x*), 2(*y*), 2(*-y*); ϕ4 = *x*; receiver = *x*,*-x*,-*x*,*x*,* -x*,*x*,*x*,*-x*. Quadrature detection in *t*_1_ is achieved using the Rance-Kay gradient selection scheme (Kay et al. 1992; Schleucher et al. 1993), with the phase ϕ4 inverted together with the gradients g6 for each complex point in *t*_1_. **B** Selected relaxation dispersion profiles obtained for the L99A mutant of T4 lysozyme at 600 and 800 MHz (5 ºC). Experimental data are shown with open circles; continuous solid lines represent best-fits to the 2-state exchange model. The upper pair of profiles (shown with dark blue and magenta lines for 600 and 800 MHz, respectively) was recorded using the fully decoupled NH_2_-HSQC based CPMG experiment of (Mulder et al. 2001), while the lower pair of profiles (shown with light blue and red lines for 600 and 800 MHz, respectively) were obtained with the experiment shown in panel (**A**). The indicated exchange parameters are averages over individual N–H correlations within NH_2_ groups and extracted from the best-fits of the NH_2_-TROSY CPMG dispersion profiles. See SI, Table S3, comparing the exchange parameters for the three sites in panel (**B**) derived from different variants of CPMG experiments

The benefits of NH_2_-TROSY can be fully realized in NMR experiments that require prolonged time intervals for relaxation/evolution of ^15^N transverse magnetization. Constant-time (CT) CPMG relaxation dispersion experiments (Mulder et al. 2000) for studies of chemical exchange on the *μ*s-to-ms timescale is one example of such NMR applications. The pulse scheme for the NH_2_-TROSY based CPMG relaxation dispersion experiment is shown in Fig. 7A, and represents a straightforward extension of the experiment in Fig. 4 with selection of the slow-relaxing ^15^N components, *N*^ββ^, before the CPMG time interval and *t*_1_ acquisition period, followed by the transfer of magnetization to the *H*^ββ^ components of H_*Z*_ and H_*E*_ protons for detection. The experiment was applied to Asn and Gln side chain NH_2_ groups of the L99A mutant of T4 lysozyme (Eriksson et al. 1992b, 1992a) at 5 ºC. The mutation at position 99 of the C-terminal domain of the protein leads to the formation of a buried cavity that binds a variety of ligands, such as substituted benzenes, indole, xenon and others (Feher et al. 1996). This mutant emerged as a model system for investigating the role of protein dynamics in ligand binding by solution techniques, as the X-ray studies show that access of ligands to the cavity is not possible in static structures (Eriksson et al. 1992b). Previous CPMG relaxation dispersion based NMR studies showed that many of Asn and Gln side chains in the L99A mutant undergo chemical exchange processes on the timescales between ~1 and ~3 ms at 25 ºC and that these motions do not occur in the wild type protein (Mulder et al. 2001).

The CPMG relaxation dispersion profiles obtained for the T4 lysozyme mutant at 5 ºC using the pulse scheme in Fig. 7A with the CT relaxation delay *T* set to 30 ms, were compared with those recorded with the decoupled NH_2_-HSQC based CPMG experiment of (Mulder et al. 2001). This relatively short relaxation delay was chosen to ensure that the weakest NH_2_ correlations are still observable in the series of 2D spectra. Although the observed gains in sensitivity of the NH_2_-TROSY experiment are very modest on average – ~ 15% (800 MHz; *t*_1,max_ = 75 ms) – several NH_2_ sites show increases in signal-to-noise ratio of up to a factor of ~2 in the NH_2_-TROSY CPMG spectra. Effective ^15^N transverse relaxation rates, *R*_2,eff_, in the limit of fast pulsing (CPMG frequency *ν*_CPMG_ → ∞), *R*_∞_, are, as expected, uniformly lower in the NH_2_-TROSY CPMG experiment—by a factor of 2.4 on average (800 MHz). Slower relaxation of the ^15^N TROSY component potentially permits using longer relaxation delays in CPMG relaxation dispersion experiments leading to better sampling of profiles and characterization of exchange processes that are slow on the chemical shift timescale. It is important to note that the element of duration 8*τ*_b_ in the middle of the two *T*/2 time periods (Fig. 7A), serves an additional purpose of eliminating the effects of cross-relaxation between different ^15^N components arising from dipolar interactions with external ^1^H spins. Although the ^15^N-^1^H_2_ TROSY component (*N*^ββ^) does not cross-relax directly with the anti-TROSY one (*N*^αα^) in ^15^N–^1^H_2_ spin-systems, it does so with the (unobservable) components *N*^αβ^ and *N*^βα^ (*cf.* the expression for the relaxation matrix $\tilde{\Gamma }_{{{\text{HH}}}}^{{{\text{ext}}}}$ in SI, Eq. S3), which have much faster relaxation rates. The element of duration 8*τ*_b_ effectively inverts the ^15^N TROSY component in the middle of the relaxation interval *T* (note that the transitions *N*^αβ^ and *N*^βα^ do not evolve from ^1^*J*_NH_ couplings) eliminating to first order the effects of cross-relaxation on the *R*_2,eff_ rates (Loria et al. 1999) in the limit of fast pulsing (high ν_CPMG_ fields; when the refocusing pulses of the CPMG pulse train are applied rapidly compared to 1/(2^1^*J*_NH_), thereby suppressing scalar coupling evolution). The utility of the element of duration 8*τ*_b_ for elimination of the effects of cross-relaxation in CPMG experiment applied to NH_2_ groups, is discussed at length in (Mulder et al. 2001), and is analogous to the role of selective inversion pulses applied during mixing periods of NOESY experiments to reverse cross-relaxation induced transfer of magnetization (Vincent et al. 1996).

The NH_2_-TROSY CPMG relaxation dispersion profiles recorded at 600 and 800 MHz (5 ºC) with *R*_ex_ >  ~3 s^−1^ are compared with those obtained with the NH_2_–HSQC based CPMG experiment (Mulder et al. 2001) in Fig. 7B. Consistently lower *R*_2,eff_ rates are observed in the NH_2_-TROSY CPMG data (lower sets of profiles). Note also somewhat lower *R*_∞_ rates for the NH_2_-TROSY profiles at 800 MHz (shown in red) compared to those recorded at 600 MHz (light blue), reflecting more efficient cancellation of dipolar fields by the ^15^N CSA/N–H DD cross-correlations at a higher *B*_0_ field (Fig. 3). The parameters of exchange — the timescale, *τ*_ex_, the fractional population of the minor state, *p*_B_, and the chemical shift difference between the inter-converting species, Δω — were extracted from the best-fits of dispersion profiles at the two spectrometer fields to a 2-state model of exchange for each individual N–H correlation. As expected, very similar exchange parameters were obtained between the two N–H correlations within each NH_2_ group, as well as between the same NH_2_ sites in the NH_2_-TROSY and NH_2_-HSQC CPMG experiments. The values reported in Fig. 7B are averages over the parameters obtained for the two N–H correlations of each NH_2_ site in the NH_2_-TROSY CPMG experiment. Note that the inter-conversion between the major and minor conformational species for Asn132 (the rightmost panel in Fig. 7B) becomes too slow on the chemical shift timescale at 5 ºC for reliable derivation of individual parameters, and only the ratio *p*_B_/*τ*_ex_ can be extracted with confidence. Table S3 in the SI compares the exchange parameters for the three NH_2_ sites in Fig. 7B derived from the HSQC-based and TROSY-based CPMG relaxation dispersion profiles recorded at 5 ºC, as well as the exchange parameters for the same NH_2_ sites obtained from the HSQC-based relaxation dispersion profiles obtained earlier by (Mulder et al. 2001) and in this work at 25 ºC. We note that for 2 out of the 3 NH_2_ sites shown in Fig. 7B (Asn140 and Asn132), some of the exchange parameters could not be determined from the best-fits of the NH_2_-HSQC CPMG derived profiles (upper curves in Fig. 7B; see Table S3) indicating that the information content of the CPMG relaxation dispersion profiles increases with slower intrinsic relaxation of the ^15^N NH_2_-TROSY components. Although the number of NH_2_ probes with significant relaxation dispersion at 5 ºC is limited to only 3 sites, comparison of exchange parameters between the two temperatures (SI, Table S3), indicates that a large increase in the fractional population of minor species occurs upon decrease in temperature from 25 to 5 ºC, while no major changes in Δω values are observed between the two temperatures.

## Concluding remarks

We have developed a transverse relaxation optimized spectroscopy (TROSY) based approach for optimal detection of NH_2_ groups in asparagine and glutamine side chains of proteins. NMR experiments for the isolation of the slow-relaxing components of NH_2_ multiplets are described. Although even modest sensitivity gains in 2D NH_2_-TROSY correlation maps compared to their decoupled NH_2_-HSQC counterparts can be achieved only infrequently, substantial improvements in resolution of NMR spectra are demonstrated for aparagine and glutamine NH_2_ sites in a buried cavity mutant L99A of T4 lysozyme at 5 ºC. The NH_2_-TROSY approach is applied to CPMG relaxation dispersion measurements at the side chain NH_2_ positions of the L99A T4 lysozyme mutant—a model system for studies of the role of protein dynamics in ligand binding.

## Materials and methods

### NMR sample

The U-[^15^N]-labeled sample of T4 lysozyme, containing mutations C54T/C97A/L99A, was prepared closely following the protocol described previously (Matsumura et al. 1988). The protein was expressed in BL21DE3 *E. coli* cells harboring the pHS1403 plasmid (Addgene #18,476), and purified by anion exchange chromatography using a Sepharose High Performance (SP HP) column, with elution achieved by a NaCl gradient. The purified lysozyme fractions were subjected to gel filtration using a Superdex 75 column. The sample concentration was 0.9 mΜ in a buffer containing 90% H_2_O /10% D_2_O, 20 mM sodium phosphate, pH 6.5, and 20 mM NaCl.

### NMR spectroscopy

All NMR spectra were recorded on 600, 800 and 900 MHz AVANCE HD (600) or NEO (800 and 900) Bruker spectrometers equipped with triple-axis (*x*, *y*, *z*) gradient cryogenic probes at 5 and 25 ºC. The spectra were processed and analyzed using the NMRPipe/NMRDraw programs and associated software (Delaglio et al. 1995) and plotted with the NMRView program (Johnson and Blevins 1994). The assignments of NH_2_ groups in the L99A mutant of T4 lysozyme were taken from (Mulder et al. 2001). Each of the 2D spectra acquired with the pulse scheme in Fig. 4 and the 2D HSQC spectra optimized for NH_2_ groups (Schleucher et al. 1994) (5 ºC; 900 MHz) comprised [64*; 700*] complex points in the [^15^N(*t*_1_); ^1^H(*t*_2_)] dimensions translating to acquisition times of [88; 64] ms. Typically, 16 scans per FID were used for selection of each of the components of NH_2_ multiplet with an inter-scan recovery delay of 2.0 s, leading to net acquisition times of ~ 72 min per 2D spectrum.

The CPMG relaxation dispersion pseudo-3D series of spectra acquired on the L99A mutant of T4 lysozyme at 5 ºC using the pulse scheme in Fig. 7A and the NH_2_-HSQC based CPMG experiment of (Mulder et al. 2001) comprised [48*, 700*] complex points in the [^15^N(*t*_1_); ^1^H(*t*_2_)] dimensions translating to acquisition times of [99; 64] ms and [75; 64] ms at 600 and 800 MHz, respectively. The corresponding values for CPMG experiments recorded at 25 ºC were: [64*, 700*] complex points in the [^15^N(*t*_1_); ^1^H(*t*_2_)] dimensions translating to acquisition times of [132; 64] ms and [100; 64] ms at 600 and 800 MHz, respectively. Typically, 32(24) scans per FID with an inter-scan recovery delay of 2.2 s were used at 5(25) ºC leading to net acquisition times of ~2.0 h per 2D spectrum. The relaxation delay *T* was set to 30 ms and 50 ms for experiments at 5 and 25 ºC, respectively. The following sets of CPMG field strengths, ν_CPMG_ = 1/(4*τ*_CPMG_), with 2*τ*_CPMG_ the separation between successive refocusing pulses, were used in addition to the reference data set collected without the delay *T* (ν_CPMG_ = 0): (66.7, 133.3, 200, 266.7, 333.3, 400, 466.7, 533.3, 600, 666.7, 800, 866.7, 1000) Hz at 5 ºC, and (40, 80, 120, 160, 200, 240, 280, 320, 360, 400, 440, 480, 520, 560, 640, 720, 800, 880, 1000) Hz at 25 ºC.

### Density functional theory (DFT) calculations of ^15^N and ^1^H chemical shielding anisotropy (CSA) tensors in carboxamide NH_2_ groups

The CSA tensors of ^15^N and ^1^H nuclei of carboxamide NH_2_ groups in Asn and Gln side chains were calculated using the Gaussian 16 (Frisch et al. 2016) and ORCA (Neese et al. 2020) programs. Initial geometries of Ac-Gln-NH_2_ and Ac-Asn-NH_2_ fragments, where ‘Ac’ stands for the acetyl group, were constructed from the atomic coordinates of Gln41 and Asn25 in the 1.8 Å X-ray structure of ubiquitin (PDB entry 1UBQ (Vijay-Kumar et al. 1987)) and from Gln123 and Asn101 in the 1.8 Å X-ray structure of T4 lysozyme (PDB entry 6LZM (Eriksson et al. 1992b)). The effect of hydrogen bonding on CSA tensors was investigated by inclusion of hydrogen bond acceptors represented by acetamide fragments or water molecules from the environment of each side chain NH_2_ moiety in the corresponding X-ray structure. Among these fragments, Gln41 of ubiquitin represents a particularly interesting probe (Scheurer et al. 1999), as both of its NH_2_ hydrogens are hydrogen-bonded to the carbonyl groups of Lys27 and Ile36, allowing the effect of hydrogen bonding on CSA tensors to be investigated by including or excluding one or both of the hydrogen bond acceptors. The geometries of Ac-Gln(Asn)-NH_2_ hydrogens with and without hydrogen bond acceptors (acetamide moieties or water molecules) were first optimized using the B3LYP hybrid functional (Becke 1993) and 6–311++G** basis set (McLean and Chandler 1980; Krishnan et al. 1980), with the coordinates of all non-hydrogen atoms fixed. To account for effects of water solvation, the Solvation Model Density (SMD) solvation model (Marenich et al. 2009) was applied during all calculations using the self-consistent reaction field approach (Tomasi et al. 2005). The CSA tensors were computed from the optimized geometries using the Gauge Independent Atomic Orbital (GIAO) method (Wolinski et al. 1990) at the same level of theory, and visualized using the TensorView application (Svenningsson and Mueller 2023) implemented in Matlab®.

The CSA tensors *σ* were symmetrized by averaging the tensor with its transpose, (*σ* + *σ*^T^)/2, where ‘T’ denotes transposition. The symmetrical tensors were diagonalized yielding the principal components *σ*_*kk*_, (*k* = 1, 2, 3), as eigenvalues and the principal axes as eigenvectors. The principal components of the traceless CSA tensors, *σ*_*ii*_ (*i* = *X*, *Y*, *Z*), are then calculated as, *σ*_*ii*_ = * σ*_*kk*_* - σ*_*iso*_, where *σ*_*iso*_ = (*σ*_11_ + *σ*_22_ + *σ*_33_)/3. The largest by absolute magnitude component of the traceless CSA tensor is chosen as *σ*_*ZZ*_ following the Haeberlen convention (Haeberlen 1976) (|*σ*_*ZZ*_| ≥|*σ*_*XX*_ | ≥|*σ*_*YY*_ |). The anisotropy of the CSA tensors, Δσ, is calculated as, *σ*_*ZZ*_ - (*σ*_*XX*_ + *σ*_*YY*_)/2.

The principal values and orientations of the ^15^N CSA tensor obtained from the calculation with the program Gaussian for non-hydrogen and hydrogen-bonded side chain NH_2_ group of Gln41 in ubiquitin are reported in the SI, Table S1. The principal values and orientations of the non-hydrogen and hydrogen-bonded ^1^H_*E*_ and ^1^H_*Z*_ CSA tensors obtained for the same NH_2_ site of ubiquitin (Gaussian) are listed in the SI, Table S2. DFT calculations showed that the CSA tensors of both ^15^N and ^1^H nuclei may be significantly asymmetric—particularly for hydrogens (Yao et al. 2010a, 2010b), with asymmetry *η* = (*σ*_*YY*_–*σ*_*XX*_)/*σ*_*ZZ*_ for ^1^H CSA tensors varying from 0 to 0.95 depending on the environment. For the purposes of simplification, we assume axial symmetry of CSA tensors in the ‘Results and Discussion’ section and provide exact expressions for auto- and cross correlated relaxation rates derived by (Goldman 1984), in the SI.

## Supplementary Information

Below is the link to the electronic supplementary material.

Supplementary file1 (PDF 571 KB)—Expansion of the expressions in Eqs. 1 and 4 for ^15^N and ^1^H multiplet components of NH_2_ groups. Full relaxation matrices describing the evolution of ^15^N and ^1^H transitions in NH_2_ spin-systems due to transverse relaxation calculated in the macromolecular limit. Three tables listing the principal values and orientations of the ^15^N and ^1^H_*Z/E*_ CSA tensors obtained from DFT calculations for the side chain NH_2_ group of Gln41 in the protein ubiquitin (Tables S1-S2) and the parameters of exchange at the NH_2_ sites of the L99A mutant of T4 lysozyme shown in Figure 7B (Table S3)

## Data Availability

No datasets were generated or analysed during the current study.
