# Supplementary material for: Transverse relaxation optimized spectroscopy of NH2 groups in glutamine and asparagine side chains of proteins
Source: J Biomol NMR. 2024 Jul 31;78(4):199–213. doi: 10.1007/s10858-024-00445-8 (PMC11614955; doi:10.1007/s10858-024-00445-8)
Supplement: Supplementary file 1 — Supplementary file1 (PDF 571 KB)—Expansion of the expressions in Eqs. 1 and 4 for 15N and 1H multiplet components of NH2 groups. Full relaxation matrices describing the evolution of 15N and 1H transitions in NH2 spin-systems due to transverse relaxation calculated in the macromolecular limit. Three tables listing the principal values and orientations of the 15N and 1HZ/E CSA tensors obtained from DFT calculations for the side chain NH2 group of Gln41 in the protein ubiquitin (Tables S1-S2) and the parameters of exchange at the NH2 sites of the L99A mutant of T4 lysozyme shown in Figure 7B (Table S3) [file 10858_2024_445_MOESM1_ESM.pdf]

Supplementary Information

Transverse relaxation optimized spectroscopy of NH_2 groups in glutamine and asparagine side chains of proteins

Vitali Tugarinov,* Francesco Torricella, Jinfa Ying, and G. Marius Clore*

Laboratory of Chemical Physics, National Institute of Diabetes and Digestive and Kidney Diseases, National Institutes of Health, Bethesda, MD 20892-0520, USA

*Authors to whom correspondence should be addressed:

G.M.C. e-mail: mariusc@mail.nih.gov; V.T. e-mail: vitali.tugarinov@nih.gov

Expansion of the expressions for ^{15}N and ^{1}H multiplet components of NH_2 groups in Eqs. 1 and 4. Transverse relaxation matrices for ^{15}N and ^{1}H transitions of NH_2 spin-systems in the macromolecular limit used for the calculations of relaxation rates.

Below we provide differential equations that describe transverse relaxation decay processes (R_2) within a $^{15}NH_2$ spin-system in the macromolecular limit (considering only $J(0)$ terms). The column vector of ^{15}N transitions, $\vec{L}$, consists of four components given by,

$$L_1\ (N^{\alpha\alpha}) = N_\pm + 2N_\pm H_{Z,1} + 2N_\pm H_{Z,2} + 4N_\pm H_{Z,1}H_{Z,2}$$

$$L_2\ (N^{\alpha\beta}) = N_\pm + 2N_\pm H_{Z,1} - 2N_\pm H_{Z,2} - 4N_\pm H_{Z,1}H_{Z,2} \qquad \text{(S1)}$$

$$L_3\ (N^{\beta\alpha}) = N_\pm - 2N_\pm H_{Z,1} + 2N_\pm H_{Z,2} - 4N_\pm H_{Z,1}H_{Z,2}$$

$$L_4\ (N^{\beta\beta}) = N_\pm - 2N_\pm H_{Z,1} - 2N_\pm H_{Z,2} + 4N_\pm H_{Z,1}H_{Z,2}$$

The evolution of $\vec{L}$ due to auto- and cross-correlated relaxation processes in $^{15}NH_2$ groups is described by,

$$d\vec{L}/dt = -\left(\tilde{\Gamma}^{\mathrm{DD}}_{\mathrm{NH_1}} + \tilde{\Gamma}^{\mathrm{DD}}_{\mathrm{NH_2}} + \tilde{\Gamma}^{\mathrm{CSA}}_{\mathrm{N}} + \tilde{\Gamma}^{\mathrm{DD,DD}}_{\mathrm{NH_1,NH_2}} + \tilde{\Gamma}^{\mathrm{CSA,DD}}_{\mathrm{N,NH_1}} + \tilde{\Gamma}^{\mathrm{CSA,DD}}_{\mathrm{N,NH_2}} + \tilde{\Gamma}^{\mathrm{DD}}_{\mathrm{HH}} + \tilde{\Gamma}^{\mathrm{ext}}_{\mathrm{HH}}\right)\vec{L} \qquad \text{(S2)}$$

where $\tilde{\Gamma}^{j}_{\mathrm{A(M)}}$ and $\tilde{\Gamma}^{j,k}_{\mathrm{A(M),AX}}$ are auto- and cross-correlated relaxation matrices for nuclei A(M), respectively, due to relaxation mechanisms j, k (CSA, DD). The explicit form of Eq. S2 is given by,

$$\frac{d}{dt}\begin{bmatrix} L_1 \\ L_2 \\ L_3 \\ L_4 \end{bmatrix} = -\left(\begin{array}{l} \frac{2}{5}\left(k_{\mathrm{NH}}^{\mathrm{DD}}\right)^2 S^2\tau_C \begin{pmatrix} 1 & 0 & 0 & 0 \\ 0 & 1 & 0 & 0 \\ 0 & 0 & 1 & 0 \\ 0 & 0 & 0 & 1 \end{pmatrix} + \frac{4}{45}\left(k_{\mathrm{N}}\Delta\sigma_{\mathrm{N}}\right)^2\left(1+\frac{\eta^2}{3}\right)S^2\tau_C \begin{pmatrix} 1 & 0 & 0 & 0 \\ 0 & 1 & 0 & 0 \\ 0 & 0 & 1 & 0 \\ 0 & 0 & 0 & 1 \end{pmatrix} + \\ \frac{2}{5}\left(k_{\mathrm{NH}}^{\mathrm{DD}}\right)^2 P_2\left(\cos\theta_{\mathrm{NH_1,NH_2}}^{\mathrm{DD,DD}}\right)S^2\tau_C \begin{pmatrix} 1 & 0 & 0 & 0 \\ 0 & -1 & 0 & 0 \\ 0 & 0 & -1 & 0 \\ 0 & 0 & 0 & 1 \end{pmatrix} + \\ \frac{4}{15}k_{\mathrm{NH}}^{\mathrm{DD}}k_{\mathrm{N}} \begin{bmatrix} (\sigma_{XX}-\sigma_{ZZ})P_2\left(\cos\theta_{XX,\mathrm{NH_1}}^{\mathrm{CSA,DD}}\right) + \\ (\sigma_{YY}-\sigma_{ZZ})P_2\left(\cos\theta_{YY,\mathrm{NH_1}}^{\mathrm{CSA,DD}}\right) \end{bmatrix} S^2\tau_C \begin{pmatrix} 1 & 0 & 0 & 0 \\ 0 & 1 & 0 & 0 \\ 0 & 0 & -1 & 0 \\ 0 & 0 & 0 & -1 \end{pmatrix} + \\ \frac{4}{15}k_{\mathrm{NH}}^{\mathrm{DD}}k_{\mathrm{N}} \begin{bmatrix} (\sigma_{XX}-\sigma_{ZZ})P_2\left(\cos\theta_{XX,\mathrm{NH_2}}^{\mathrm{CSA,DD}}\right) + \\ (\sigma_{YY}-\sigma_{ZZ})P_2\left(\cos\theta_{YY,\mathrm{NH_2}}^{\mathrm{CSA,DD}}\right) \end{bmatrix} S^2\tau_C \begin{pmatrix} 1 & 0 & 0 & 0 \\ 0 & -1 & 0 & 0 \\ 0 & 0 & 1 & 0 \\ 0 & 0 & 0 & -1 \end{pmatrix} + \\ \frac{1}{10}\left(k_{\mathrm{HH}}^{\mathrm{DD}}\right)^2 S^2\tau_C \begin{pmatrix} 0 & 0 & 0 & 0 \\ 0 & 1 & -1 & 0 \\ 0 & -1 & 1 & 0 \\ 0 & 0 & 0 & 0 \end{pmatrix} + \frac{1}{10}\left(k_{\mathrm{HH}}^{\mathrm{ext}}\right)^2 \tau_C \begin{pmatrix} 1 & -1/2 & -1/2 & 0 \\ -1/2 & 1 & 0 & -1/2 \\ -1/2 & 0 & 1 & -1/2 \\ 0 & -1/2 & -1/2 & 1 \end{pmatrix} \end{array} \right) \begin{bmatrix} L_1 \\ L_2 \\ L_3 \\ L_4 \end{bmatrix} \quad \text{(S3)}$$

where the order of the matrices is the same as in Eq. S2; $k_{\mathrm{NH}}^{\mathrm{DD}} = -\left(\mu_0/4\pi\right)\hbar\gamma_{\mathrm{N}}\gamma_{\mathrm{H}}r_{\mathrm{NH}}^{-3}$; $k_{\mathrm{N}} = \gamma_{\mathrm{N}}B_0$; $k_{\mathrm{HH}}^{\mathrm{ext}} = -\left(\mu_0/4\pi\right)\hbar\gamma_{\mathrm{H}}^2 r_{\mathrm{HHext}}^{-3}$; μ_0 is the vacuum permeability constant, γ_i is the gyromagnetic ratio of spin i, $i \in \{\mathrm{N, H}\}$; r_{AX} and r_{HHext} are the A-X and H-H$_{\mathrm{ext}}$ inter-nuclear distances, respectively; $\Delta\sigma_{\mathrm{N}}$ is the ^{15}N chemical shielding anisotropy (CSA), $\sigma_{ZZ} - (\sigma_{XX} + \sigma_{YY})/2$, where σ_{ii} ($i = X, Y, Z$) are principal components of the ^{15}N CSA tensor; η is the asymmetry of the ^{15}N CSA tensor, $(\sigma_{YY} - \sigma_{XX})/\sigma_{ZZ}$; B_0 is the static magnetic field; τ_C is the global molecular rotational correlation time (assumed isotropic); S is the generalized order parameter (assumed the same for all types of interactions and unity for interactions with external ^{1}H spins); and $P_2(\cos(\theta_{\mu,\nu})) = (1/2)[3\cos^2(\theta_{\mu,\nu}) - 1]$, where $\theta_{\mu,\nu}$ is the angle formed between the

principal axes of interactions μ and ν. For example, $\theta^{\mathrm{CSA,DD}}_{XX,\mathrm{NH}_j}$ denotes the angle formed between the XX axis of the ^{15}N CSA tensor (σ_{XX}) and the N-H bond vector of proton j, (j = 1, 2).

The vector of ^{1}H transitions of each individual proton in NH_2 groups (H_1 is chosen here), $\vec{L}_{\mathrm{H}}$, comprises four components given by,

$$L_{1,\mathrm{H}}\ (H^{\alpha\beta}) = H_{\pm,1} - 2H_{\pm,1}N_Z - 2H_{\pm,1}H_{Z,2} + 4H_{\pm,1}H_{Z,2}N_Z$$
$$L_{2,\mathrm{H}}\ (H^{\alpha\alpha}) = H_{\pm,1} - 2H_{\pm,1}N_Z + 2H_{\pm,1}H_{Z,2} - 4H_{\pm,1}H_{Z,2}N_Z \qquad \text{(S4)}$$
$$L_{3,\mathrm{H}}\ (H^{\beta\beta}) = H_{\pm,1} + 2H_{\pm,1}N_Z - 2H_{\pm,1}H_{Z,2} - 4H_{\pm,1}H_{Z,2}N_Z$$
$$L_{4,\mathrm{H}}\ (H^{\beta\alpha}) = H_{\pm,1} + 2H_{\pm,1}N_Z + 2H_{\pm,1}H_{Z,2} + 4H_{\pm,1}H_{Z,2}N_Z$$

The evolution of $\vec{L}_{\mathrm{H}}$ due to auto- and cross-correlated relaxation processes in $^{15}NH_2$ groups during t_2 is described by,

$$d\vec{L}_{\mathrm{H}} / dt = -\left(\tilde{\Gamma}^{\mathrm{DD}}_{\mathrm{HN}} + \tilde{\Gamma}^{\mathrm{DD}}_{\mathrm{HH}} + \tilde{\Gamma}^{\mathrm{CSA}}_{\mathrm{H}} + \tilde{\Gamma}^{\mathrm{DD,DD}}_{\mathrm{HN,HH}} + \tilde{\Gamma}^{\mathrm{CSA,DD}}_{\mathrm{H,HN}} + \tilde{\Gamma}^{\mathrm{CSA,DD}}_{\mathrm{H,HH}} + \tilde{\Gamma}^{\mathrm{ext}}_{\mathrm{HH}}\right)\vec{L}_{\mathrm{H}} \qquad \text{(S5)}$$

The explicit form of Eq. S5 is given by,

$$\frac{d}{dt}\begin{bmatrix} L_{1,\mathrm{H}} \\ L_{2,\mathrm{H}} \\ L_{3,\mathrm{H}} \\ L_{4,\mathrm{H}} \end{bmatrix} = -\left(\begin{array}{l} \frac{1}{5}\left(k_{\mathrm{HN}}^{\mathrm{DD}}\right)^2 S^2\tau_C \begin{pmatrix} 1 & 0 & 0 & 0 \\ 0 & 1 & 0 & 0 \\ 0 & 0 & 1 & 0 \\ 0 & 0 & 0 & 1 \end{pmatrix} + \frac{1}{4}\left(k_{\mathrm{HH}}^{\mathrm{DD}}\right)^2 S^2\tau_C \begin{pmatrix} 1 & 0 & 0 & 0 \\ 0 & 1 & 0 & 0 \\ 0 & 0 & 1 & 0 \\ 0 & 0 & 0 & 1 \end{pmatrix} + \\ \frac{4}{45}\left(k_{\mathrm{H}}\Delta\sigma_{\mathrm{H}}\right)^2 \left(1+\frac{\eta^2}{3}\right) S^2\tau_C \begin{pmatrix} 1 & 0 & 0 & 0 \\ 0 & 1 & 0 & 0 \\ 0 & 0 & 1 & 0 \\ 0 & 0 & 0 & 1 \end{pmatrix} + \\ \frac{2}{5}\left(k_{\mathrm{HN}}^{\mathrm{DD}}\right)\left(k_{\mathrm{HH}}^{\mathrm{DD}}\right) P_2\left(\cos\theta_{\mathrm{HN,HH}}^{\mathrm{DD,DD}}\right) S^2\tau_C \begin{pmatrix} -1 & 0 & 0 & 0 \\ 0 & 1 & 0 & 0 \\ 0 & 0 & 1 & 0 \\ 0 & 0 & 0 & -1 \end{pmatrix} + \\ \frac{4}{15} k_{\mathrm{HN}}^{\mathrm{DD}} k_{\mathrm{H}} \begin{bmatrix} (\sigma_{XX}-\sigma_{ZZ})P_2\left(\cos\theta_{XX,\mathrm{HN}}^{\mathrm{CSA,DD}}\right) + \\ (\sigma_{YY}-\sigma_{ZZ})P_2\left(\cos\theta_{YY,\mathrm{HN}}^{\mathrm{CSA,DD}}\right) \end{bmatrix} S^2\tau_C \begin{pmatrix} 1 & 0 & 0 & 0 \\ 0 & 1 & 0 & 0 \\ 0 & 0 & -1 & 0 \\ 0 & 0 & 0 & -1 \end{pmatrix} + \\ \frac{4}{15} k_{\mathrm{HH}}^{\mathrm{DD}} k_{\mathrm{H}} \begin{bmatrix} (\sigma_{XX}-\sigma_{ZZ})P_2\left(\cos\theta_{XX,\mathrm{HH}}^{\mathrm{CSA,DD}}\right) + \\ (\sigma_{YY}-\sigma_{ZZ})P_2\left(\cos\theta_{YY,\mathrm{HH}}^{\mathrm{CSA,DD}}\right) \end{bmatrix} S^2\tau_C \begin{pmatrix} -1 & 0 & 0 & 0 \\ 0 & 1 & 0 & 0 \\ 0 & 0 & -1 & 0 \\ 0 & 0 & 0 & 1 \end{pmatrix} + \\ \frac{3}{10}\left(k_{\mathrm{HH}}^{\mathrm{ext}}\right)^2 \tau_C \begin{pmatrix} 1 & -1/6 & 0 & 0 \\ -1/6 & 1 & 0 & 0 \\ 0 & 0 & 1 & -1/6 \\ 0 & 0 & -1/6 & 1 \end{pmatrix} \end{array} \right) \begin{bmatrix} L_{1,\mathrm{H}} \\ L_{2,\mathrm{H}} \\ L_{3,\mathrm{H}} \\ L_{4,\mathrm{H}} \end{bmatrix} \tag{S6}$$

where the order of the matrices is the same as in Eq. S5; $k_{\mathrm{HN}}^{\mathrm{DD}} = k_{\mathrm{NH}}^{\mathrm{DD}}$; $k_{\mathrm{HH}}^{\mathrm{DD}} = -\left(\mu_0 / 4\pi\right)\hbar\gamma_{\mathrm{H}}^2 r_{\mathrm{HH}}^{-3}$; $k_{\mathrm{H}} = \gamma_{\mathrm{H}} B_0$; $\Delta\sigma_{\mathrm{H}}$ is the ^{1}H CSA, and η, the asymmetry of the ^{1}H CSA tensor (both defined as for ^{15}N above).

Table S1. The principal values and orientations of the $^{15}N^{\varepsilon 2}$ CSA tensor obtained from the calculation with the program Gaussian for non-hydrogen-bonded and hydrogen-bonded side-chain NH_2 group of Gln^{41} in ubiquitin.[a]

$^{15}N^{\varepsilon 2}$ CSA tensor	σ_{XX} (ppm)	σ_{YY} (ppm)	σ_{ZZ} (ppm)	$\Delta\sigma$ [d] (ppm)	η [e]	α [f] (deg.)	β [g] (deg.)	$\theta^{CSA,DD}_{N,NH_E}$ [h] (deg.)	$\theta^{CSA,DD}_{N,NH_Z}$ [i] (deg.)
Non hydrogen-bonded [b]	83.0	16.6	-99.6	-152.1	0.67	10.6	3.9	22.8	39.3
Hydrogen-bonded [c]	73.8	27.5	-101.4	-149.4	0.45	11.6	4.9	23.5	39.2

[a] All the σ_{ii} values ($i = X, Y, Z$) are reported for the traceless CSA tensor; all the definitions are as in the main text unless mentioned below. [b] None of the protons of the NH_2 group are hydrogen-bonded. [c] Both *E* and *Z* protons of the NH_2 group are hydrogen-bonded (see 'Materials and Methods'). [d] Calculated as, $\sigma_{ZZ} - (\sigma_{XX} + \sigma_{YY})/2$. [e] Calculated as, $(\sigma_{YY} - \sigma_{XX})/\sigma_{ZZ}$. [f] The angle formed by the *XX* axis of the tensor with respect to the N–C′ bond vector (Fig. 2A). [g] The angle formed by the *YY* axis of the tensor with respect to the normal to the carboxamide plane (Fig. 2A). [h] The angle formed by the *ZZ* axis of the tensor with respect to the N–H_E bond vector (angle α_N in Fig. 2A). [i] The angle formed by the *ZZ* axis of the tensor with respect to the H_Z–N bond vector (angle β_N in Fig. 2A).

Table S2. The principal values and orientations of the $^{1}H_{E}$ and $^{1}H_{Z}$ CSA tensors obtained from the calculation with the program Gaussian for non-hydrogen-bonded and hydrogen-bonded side chain NH_2 group of Gln41 in ubiquitin.[a)]

^{1}H CSA tensor	σ_{XX} (ppm)	σ_{YY} (ppm)	σ_{ZZ} (ppm)	$\Delta\sigma$ [d] (ppm)	η [e]	α [f] (deg.)	$\theta_{H,HN}^{CSA,DD}$ [g] (deg.)	$\theta_{H,HH}^{CSA,DD}$ [h] (deg.)
				$^{1}H_{E}\,(H^{\varepsilon 21})$				
Non hydrogen-bonded [b]	-4.74	-0.81	5.54	8.31	0.71	-0.5	33.0	64.0
Hydrogen-bonded [c]	-7.48	-2.75	10.23	15.35	0.46	-6.5	21.1	51.3
				$^{1}H_{Z}\,(H^{\varepsilon 22})$				
Non hydrogen-bonded [b]	-3.97	-3.64	7.61	11.42	0.04	6.3	19.3	50.3
Hydrogen-bonded [c]	-7.34	-2.78	10.11	15.17	0.45	-10.1	10.5	39.8

[a] All the σ_{ii} values ($i = X, Y, Z$) are reported for the traceless CSA tensor; all the definitions are as in the main text unless mentioned below. [b] None of the protons of the NH_2 group are hydrogen-bonded. [c] Both *E* and *Z* protons of the NH_2 group are hydrogen-bonded (see 'Materials and Methods'). [d] Calculated as, $\sigma_{ZZ} - (\sigma_{XX} + \sigma_{YY})/2$. [e] Calculated as, $(\sigma_{YY} - \sigma_{XX})/\sigma_{ZZ}$. [f] The angle formed by the *ZZ* axis of the tensor with respect to the carboxamide plane (Fig. 2B). [g] The angle formed by the *ZZ* axis of the tensor with respect to the corresponding H–N bond vector (angle α_H in Fig. 2B). [h] The angle formed by the *ZZ* axis of the tensor with respect to the H_Z–H_E vector (angle β_H in Fig. 2B).

Table S3. Exchange parameters for the NH_2 sites of the L99A T4 lysozyme mutant shown in Figure 7B, extracted from NH_2-HSQC/TROSY ^{15}N CPMG experiments acquired at 5 and 25 ºC. [a]

Experiment	p_b (%)	τ_{ex} (ms)	$\Delta\omega$ (ppm)	p_b/τ_{ex} (s^{-1})
		N140		
NH_2-HSQC CPMG (5 ºC)	- [b]	2.0 ± 0.2	0.7 ± 0.3	
NH_2-TROSY CPMG (5 ºC)	5.1 ± 1.8	2.4 ± 0.4	1.0 ± 0.2	
NH_2-HSQC CPMG (25 ºC) (this study)	1.9 ± 0.2	1.1 ± 0.1	1.5 ± 0.2	
NH_2-HSQC CPMG (25 ºC) (Mulder et al. 2001) [c]	1.6 ± 0.1	1.2 ± 0.1	1.7 ± 0.1	
		Q141		
NH_2-HSQC CPMG (5 ºC)	2.8 ± 0.2	1.8 ± 0.2	3.0 ± 0.1	
NH_2-TROSY CPMG (5 ºC)	3.2 ± 0.4	1.9 ± 0.2	2.9 ± 0.1	
NH_2-HSQC CPMG (25 ºC) (this study)	0.4 ± 0.1	1.9 ± 0.2	2.5 ± 0.1	
NH_2-HSQC CPMG (25 ºC) (Mulder et al. 2001) [c]	0.3 ± 0.1	2.0 ± 0.2	2.3 ± 0.1	
		N132 [d]		
NH_2-HSQC CPMG (5 ºC)			- [b]	- [b]
NH_2-TROSY CPMG (5 ºC)			1.8 ± 0.2	15.4 ± 2.5
NH_2-HSQC CPMG (25 ºC) (this study)	0.9 ± 0.1	2.7 ± 0.5	1.7 ± 0.2	3.3 ± 0.7
NH_2-HSQC CPMG (25 ºC) (Mulder et al. 2001) [c]	0.8 ± 0.2	2.8 ± 1.6	1.6 ± 0.3	2.9 ± 1.8

[a] The values of exchange parameters are averaged between the two N-H correlations of each NH_2 site. [b] Could not be determined from the best-fit. [c] Taken from Mulder FAA, Skrynnikov NR, Hon B, Dahlquist FW, Kay LE (2001) J Am Chem Soc 123:967-75, with the values of exchange parameters and their uncertainties rounded to the next significant digit. [d] A single (upfield in the ^1H dimension) N-H correlation was analyzed for this residue; only the ratio (p_b/τ_{ex}) could be extracted from the fits at 5 ºC.
